# Supplementary material for: Filamin A pre-mRNA editing modulates vascularization and tumor growth
Source: Mol Ther Nucleic Acids. 2022 Nov 9;30:522–34. doi: 10.1016/j.omtn.2022.11.004 (PMC9685389; doi:10.1016/j.omtn.2022.11.004)
Supplement: Document S1. Figures S1–S7 [file mmc1.pdf]

OMTN, Volume *30*

Supplemental information

Filamin A pre-mRNA editing modulates vascularization and tumor growth

Mamta Jain, Greeshma Manjaly, Kathrin Maly, Margreet R. de Vries, Michael Janisiw, Lisa König, Anne Yaël Nossent, and Michael F. Jantsch

Supplemental Information

Figure S1:

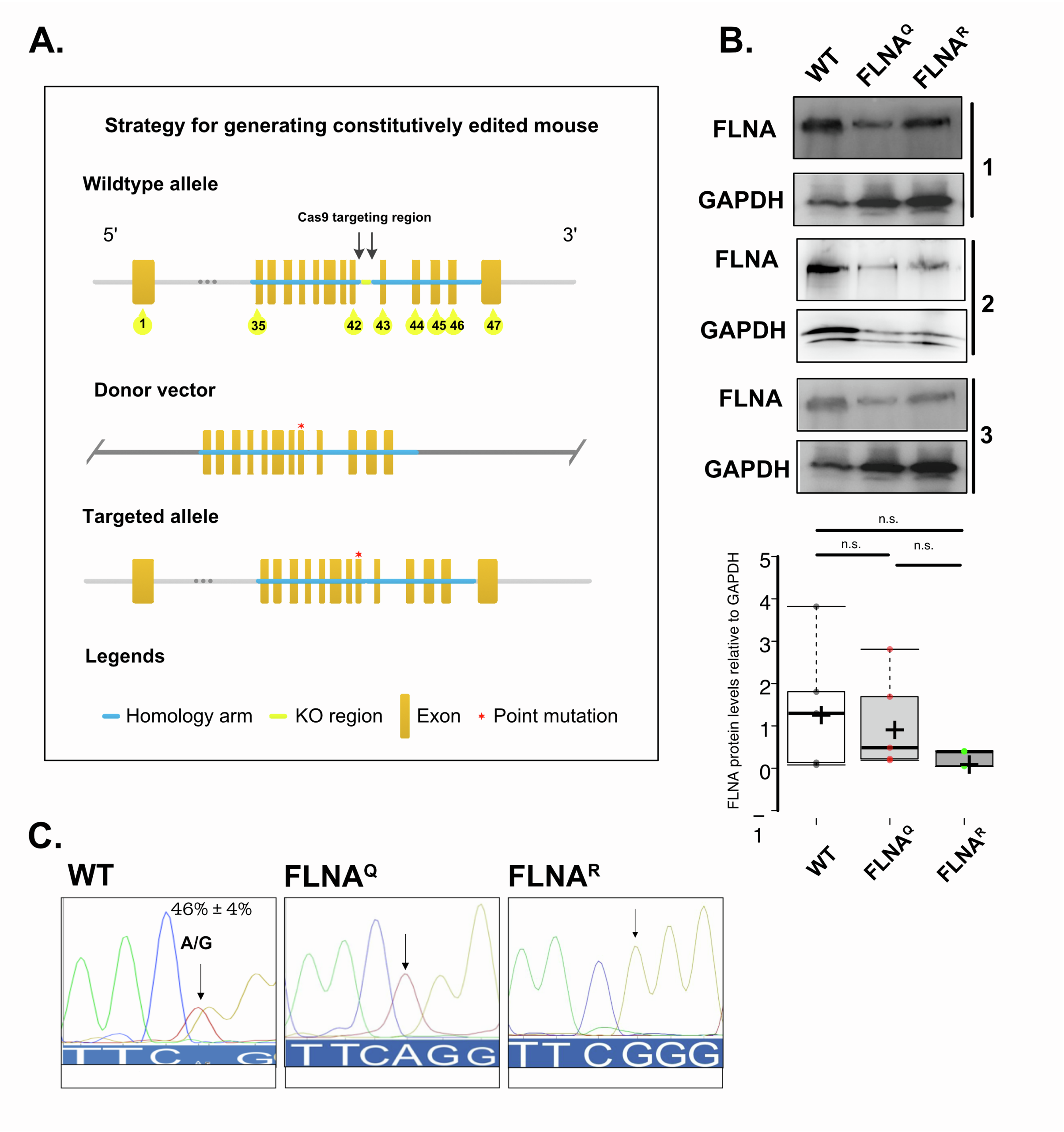

Figure S1:

Generation of transgenic mice expressing constitutively edited FLNA^{R}

(A) Scheme showing the generation of constitutively edited FLNA mouse depicting the wildtype allele, donor DNA and the targeted allele. The diagram also shows the Cas9 targeting region, deleted ECS region and the point mutation to generate a pre-edited version of FLNA. **(B)** Three Western blots showing FLNA expression in wild type (WT), FLNA^{Q} and FLNA^{R} lung tissues. GAPDH was used as a loading control. Graph shows the quantification of FLNA expression expressed as fold change difference. Data shown are mean ± SD from five

independent experiments. * $P<0.05$, ns-non-significant. **(C)** Electropherograms showing FLNA editing from cDNA of lung tissues of WT, $FLNA^Q$ and $FLNA^R$ mice. The site of editing is marked by a black arrow. WT lungs showed an average of 46% FLNA editing.

Figure S2

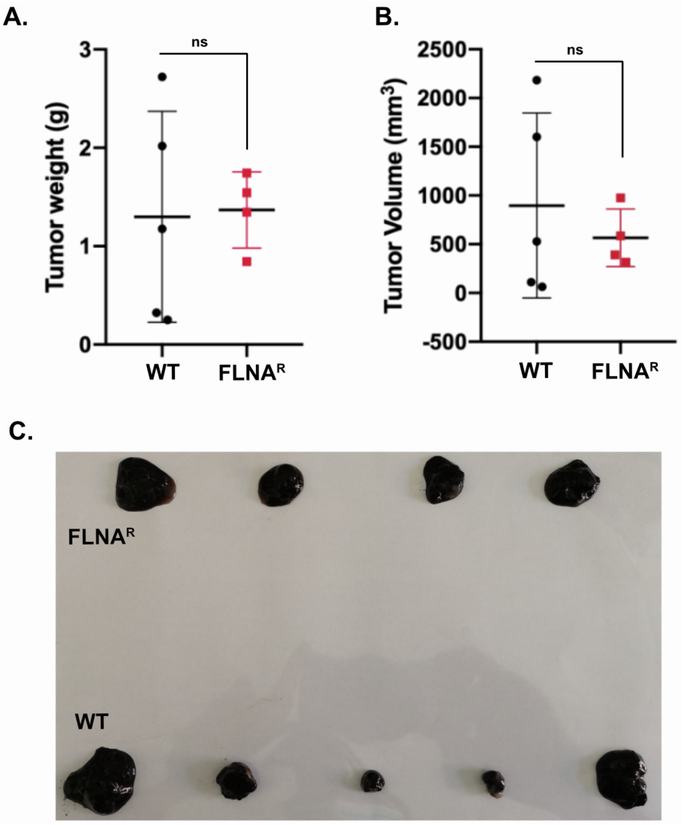

Figure S2: Comparison of growth of xenografted tumors in wild-type and transgenic mice. Graphs showing quantification of tumor weight (A) and tumor volume (B) of tumors grown in WT or transgenic mice expressing fully edited $FLNA^R$. The data represents the tumor-weight and volume measurements done on at least 4 mice per genotype. (C) shows raw images of tumors. ns: non-significant.

Figure S3:

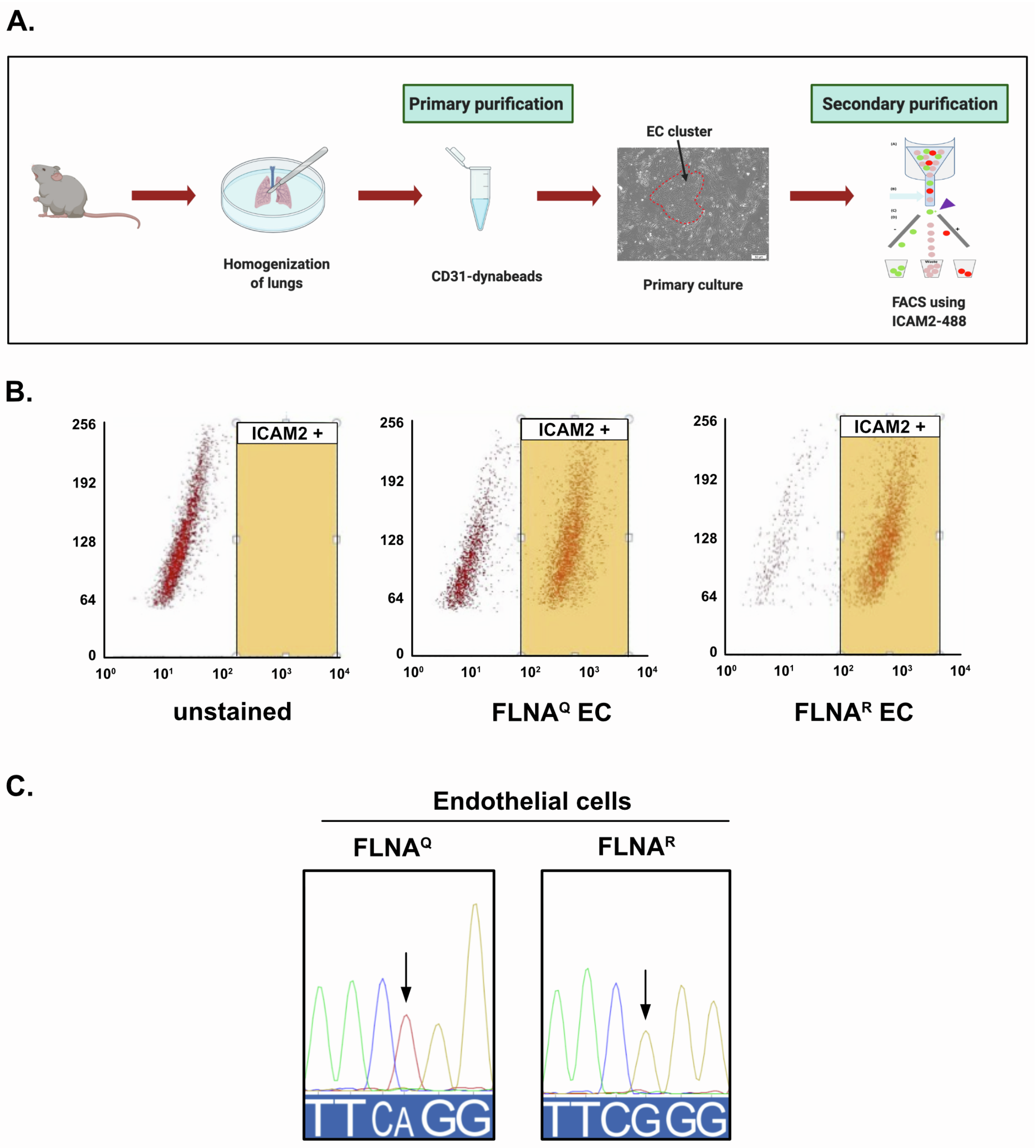

Figure S3: Purification and characterization of endothelial cells derived from mice expressing unedited FLNA^{Q} and edited FLNA^{R}

(A) Scheme depicting the steps involved in primary endothelial cell isolation from lungs of FLNA^{Q} and FLNA^{R} mice. Purification using CD31 dynabeads is followed by a second FACS-sorting step using ICAM2 antibody to obtain a pure endothelial cell population. **(B)** Graphs showing the representative FACS sorting data in unstained, FLNA^{Q} and FLNA^{R} samples. The yellow box represents the sorted endothelial cell population. **(C)** Sanger-sequencing traces showing FLNA editing on cDNA of FLNA^{Q} and FLNA^{R} endothelial cells. The site of editing is marked by a black arrow.

FigureS4:

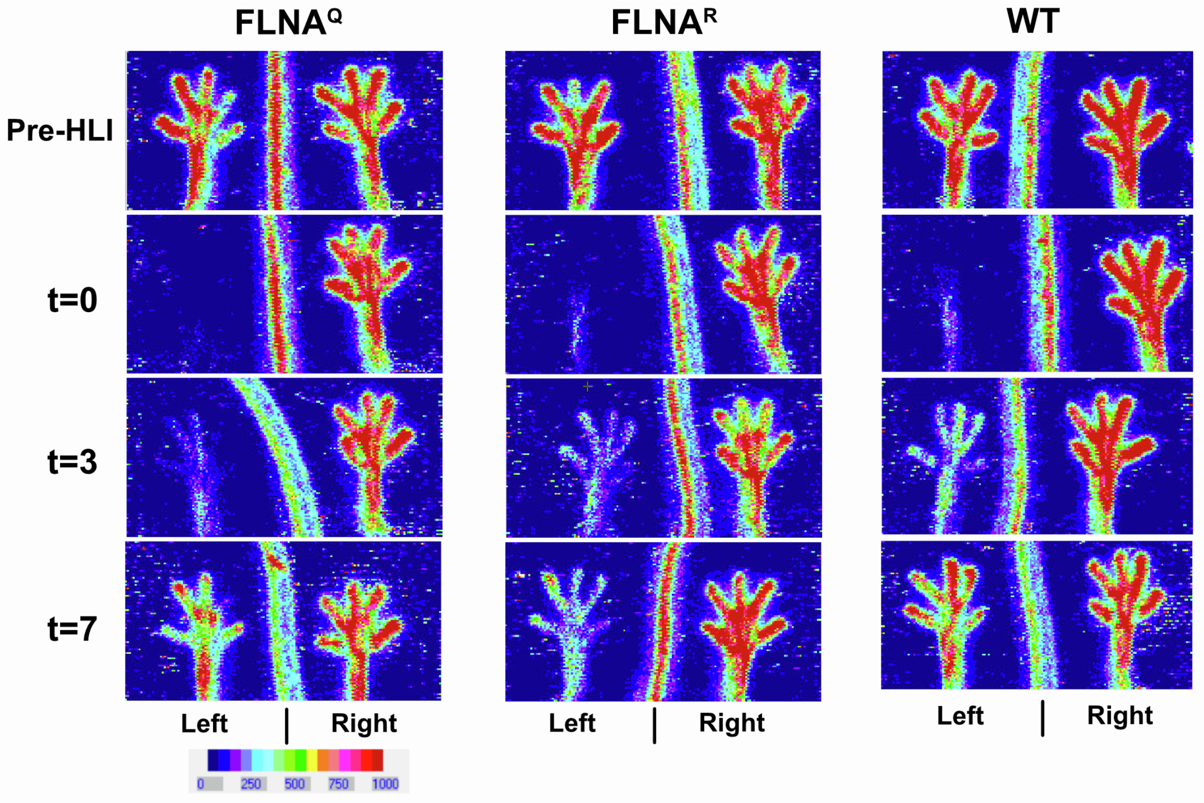

Figure S4: Reduced blood flow after induced hindlimb ischemia in mice expressing edited FLNA^R

Representative images showing blood flow recovery in the left (post-ischemic) and right control paw in FLNA^Q, FLNA^R and WT mice using Laser Doppler perfusion imaging. Paw perfusion was measured in both left (ligated) and right (control) paw pre HLI surgery, immediately after ligation (t=0) and then at 3 days (t=3) and 7 days after surgery (t=7). The shift from blue to red in color indicates an increase in blood flow. Wild-type and transgenic mice expressing unedited FLNA^Q show comparable recovery rates after seven days, while mice expressing edited FLNA^R show much reduced blood flow at 7 days post-surgery. This is an expanded figure of Figure 4 containing images of pre Hind Limb Ischemia (Pre-HLI) and 3 hrs post HLI (t=3). The images of t=0 and t=7 are therefore identical to those of Figure 4.

Figure S5:

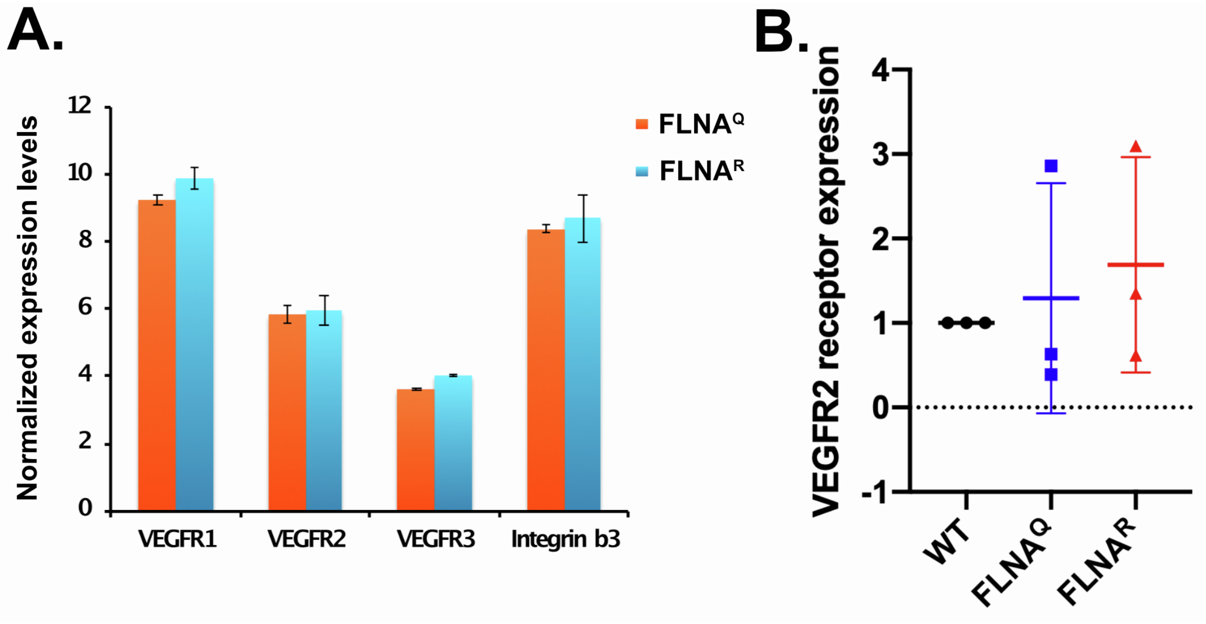

Figure S5: Expression levels of VEGFR isoforms is unchanged in FLNA^{Q} and FLNA^{R} expressing cells and mice.

(A) Expression levels of VEGFR1, VEGFR2, VEGFR3, and integrin ß1 was compared in endothelial cells expressing unedited FLNA^{Q} and edited FLNA^{R} using q-RT-PCR.
(B) Quantification of stainings of xenograft tumors grown in wild-type, FLNAQ and FLNAR expressing mice using an anti-VEGFR2 extracellular domain-specific antibody reveals equal expression of VEGFR2 at steady-state levels.

Figure S6

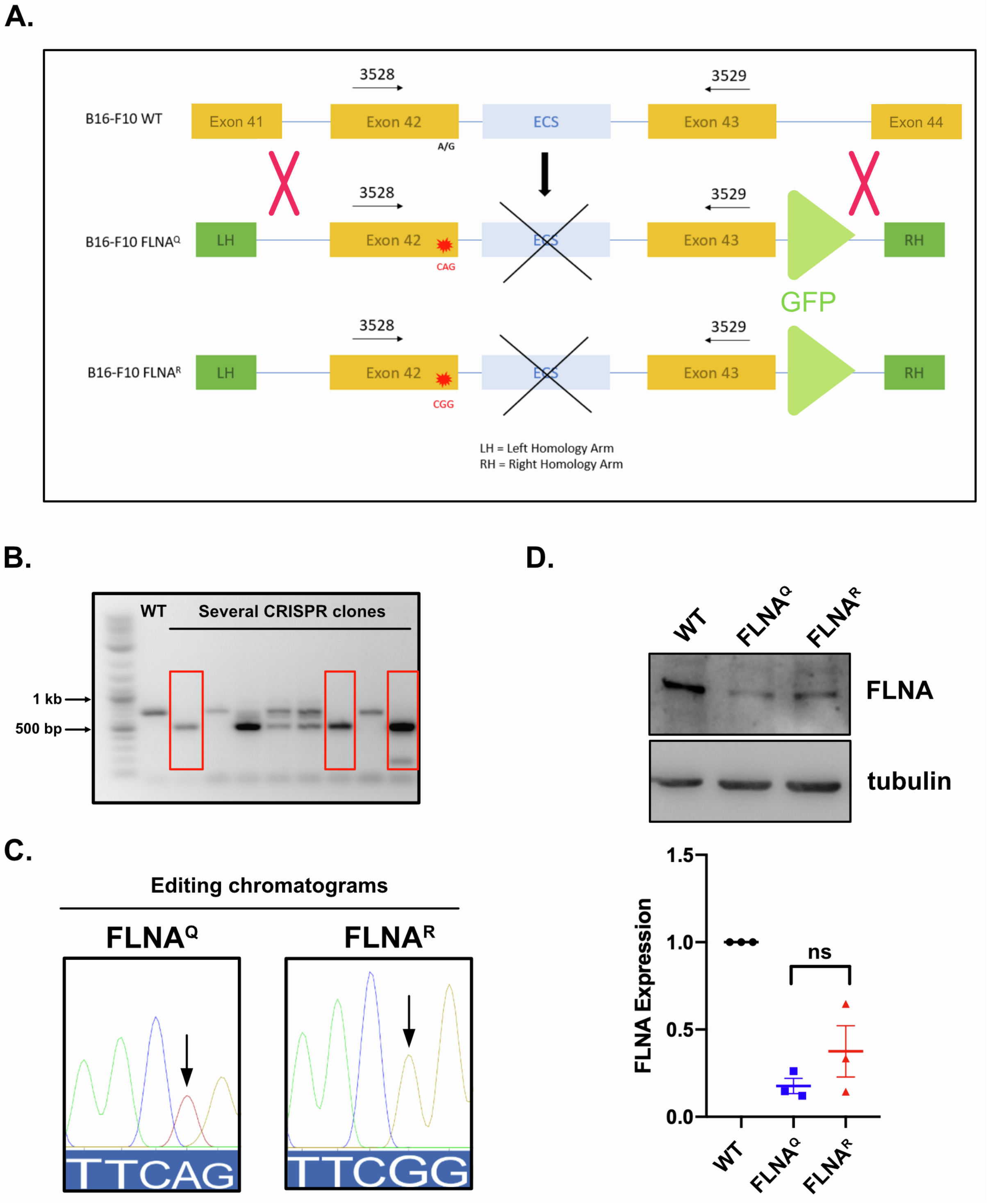

Figure S6: Generation of B16 cells expressing pre-edited FLNA^R and uneditable FLNA^Q

A) The X-linked *Flna* gene was targeted by CRISPR/Cas9 using two guide-RNAs. As a repair template a region covering parts of the gene was introduced that deleted the editing complementary sequence. In addition, one of the repair templates carried a "pre-edited" CGG codon. B) positive clones were selected by PCR by screening for the deletion of the editing complementary site. C) Expression of unedited FLNA^Q and pre-edited FLNA^R was verified by amplification of cDNA followed by Sanger sequencing of the amplification product. Exclusive expression of unedited FLNA^Q or

edited FLNA^R could be verified. D) Expression of edited and unedited FLNA was tested by western blotting and normalized to tubulin expression. Both modified alleles showed reduced expression when compared to the wild-type alle. However, the difference between edited and unedited FLNA were non-significant (ns).

Figure S7:

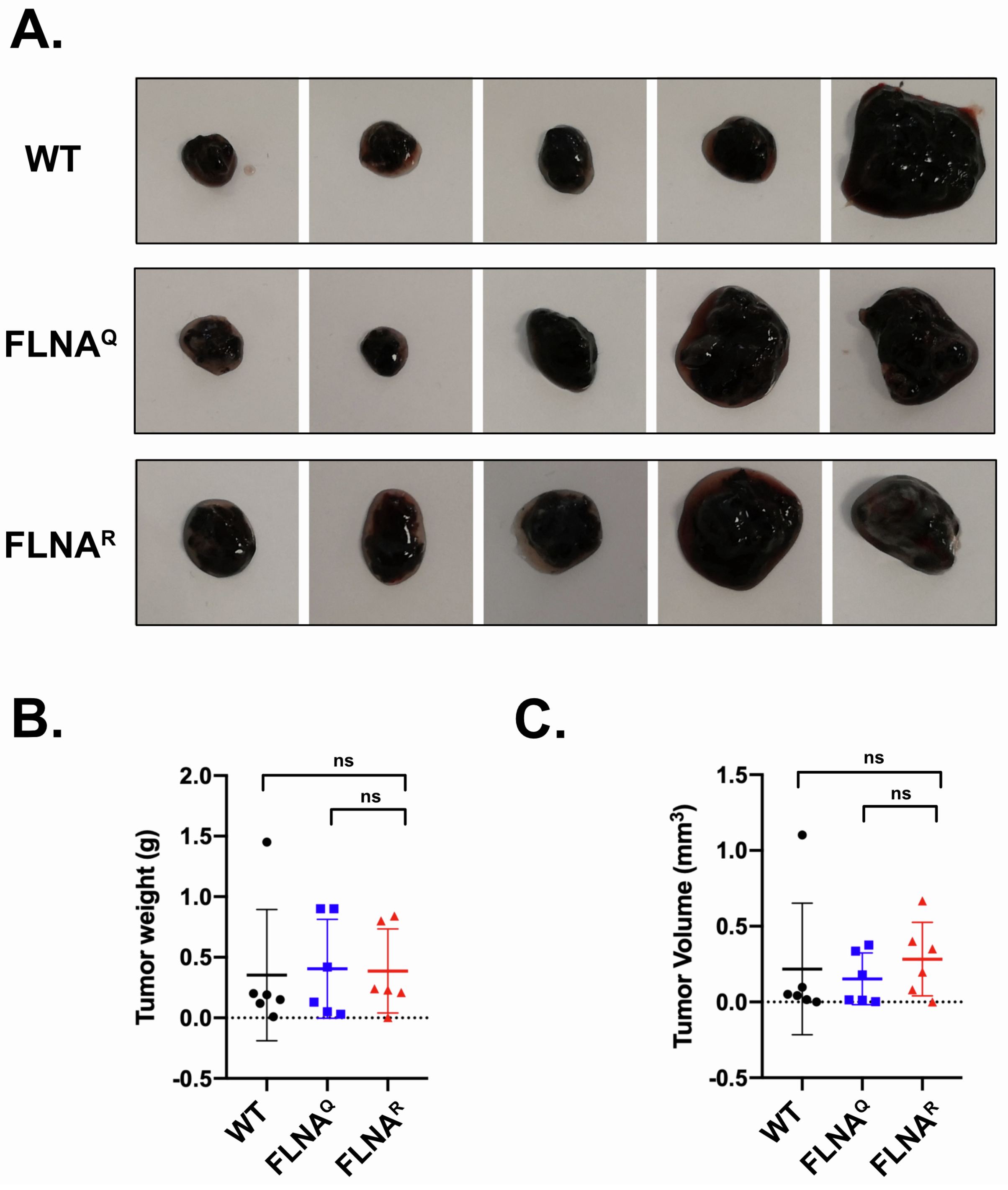

Figure S7: The editing status of B16 melanoma cells does not affect subcutaneous xenografted tumor growth (A) Images showing xenograft tumors in WT mice upon subcutaneous injections of WT, FLNA^{Q} and FLNA^{R} B16 CRISPR clones. **(B, C)** Graphs showing the quantification of tumor weight (A) and tumor volume (B) compared amongst WT, FLNA^{Q} and FLNA^{R} B16 CRISPR clones when injected in WT C57Bl6 mice subcutaneously. The data represents the mean ± SD from atleast 12 mice per genotype. ns: non-significant
