# Supplementary material for: Filamin A pre-mRNA editing modulates vascularization and tumor growth
Source: Mol Ther Nucleic Acids. 2022 Nov 9;30:522–34. doi: 10.1016/j.omtn.2022.11.004 (PMC9685389; doi:10.1016/j.omtn.2022.11.004)
Supplement: Document S2. Article plus supplemental information [file mmc2.pdf]

Molecular Therapy
Nucleic Acids
Original Article

# Filamin A pre-mRNA editing modulates vascularization and tumor growth

Mamta Jain,[1] Greeshma Manjaly,[1] Kathrin Maly,[1] Margreet R. de Vries,[2] Michael Janisiw,[1] Lisa König,[1] Anne Yaël Nossent,[2] and Michael F. Jantsch[1]

[1]Medical University of Vienna, Center of Anatomy and Cell Biology, Division of Cell and Developmental Biology, Schwarzspanierstrasse 17, 1090 Vienna, Austria; [2]Leiden University Medical Center, Department of Surgery, Einthoven Laboratory for Experimental Vascular Medicine, PO Box 9600, 2300 RC Leiden, the Netherlands

**Adenosine to inosine (A to I) editing is mediated by adenosine deaminases acting on RNA (ADAR) enzymes. Inosines are interpreted as guanosines by the translational machinery. Consequently, A to I editing in mRNAs can lead to their recoding and the formation of proteins not encoded in the genome. Filamin A is an actin-crosslinking protein. A to I editing in the filamin pre-mRNA leads to the exchange of a glutamine to an arginine in a highly interactive domain of the protein. However, the consequences of this editing event are still poorly understood. Here we show, using transgenic mice expressing either constitutively edited or constitutively uneditable filamin A that filamin A editing critically controls angiogenesis in tumors but also in a mouse ischemia model. Hyper-editing reduces angiogenesis, while hypoediting leads to increased angiogenesis, possibly by altering vascular endothelial growth factor receptor 2 (VEGFR2) turnover. Further, FLNA editing of the tumor itself seemingly affects its metastatic potential by changing its interaction with the extracellular matrix. We therefore identify filamin A editing as a critical component for angiogenesis, tumor growth, and metastasis formation.**

## INTRODUCTION

Adenosine deaminases acting on RNA (ADARs) convert adenosine to inosine (A to I) in structured and double-stranded RNAs.[1] As inosines are primarily interpreted as guanosines by cellular machineries, A to I editing can have a large impact on many cellular functions. On the one hand, A to I editing seems to mark endogenous repeat-derived RNAs as "self," thereby preventing inadvertent activation of the cytoplasmic double-stranded RNA sensor MDA5 and subsequent interferon signaling.[2] RNA-editing events can also alter splice patterns or change the proteins binding to RNAs.[1] Editing within coding regions of RNAs can even lead to the recoding of RNAs, thereby leading to the formation of proteins that are not encoded in the genome.[1] In mammals, two active ADAR enzymes can be found: *Adar* (ADAR1) and *Adarb1* (ADAR2). While ADAR1 is primarily targeting non-coding, repeat-derived RNAs, the majority of protein recoding events are mediated by ADAR2.[3] ADAR2 was first identified to be critically involved in the recoding of mammalian glutamate receptor subunit *Gria2*, where editing leads to the exchange of a CAG-glutamate (Q) to CIG-arginine (R) codon.[4] This amino acid exchange critically regulates $Ca^{2+}$ influx into cells. Mice lacking this Q to R exchange in Gria2 suffer from epilepsy and die within 3 weeks after birth.[5] Meanwhile, more than a 100 editing-induced amino acid exchanges have been characterized.[6] However, the precise impact on protein function and their impact on cellular and organismic physiology has only been investigated in very few cases.

An A to I RNA editing that is highly conserved in mammals has been identified in the pre-mRNA encoding filamin A (FLNA).[7] FLNA is built of 24 immunoglobulin (Ig)-like repeats. Editing occurs in exon 42 encoding parts of Ig-repeat 22.[8] The editing events lead to the exchange of a Q to an R amino acid exchange in a highly interactive region. FLNA editing is mainly mediated by ADAR2 and has been shown to be highest in the vasculature, the large intestine, and the stomach.[9]

Mutations in human *FLNA* lead to neuronal and intestinal diseases.[10,11] *FLNA*-deficient mice are embryonic lethal, showing severe cardiovascular defects and irregular vascular patterning.[12] Deletion of *FLNB* in mice leads to skeletal malformations.[13] FLNA is known to play a dual role in tumorigenesis. FLNA can get proteolytically cleaved, leading to differential subcellular localization of the resulting fragments. Full-length, cytoplasmic FLNA could favor tumor progression, whereas nuclear FLNA may inhibit tumorigenesis.[14,15] An endothelial-specific knockout of FLNA results in altered cardiac remodeling, reduced capillary formation, and dysregulated endothelial signaling upon induced infarction.[16] Moreover, small interfering RNA (siRNA)-mediated knockdown of *FLNA* in human umbilical vein endothelial cells (HUVECs) leads to reduced tube formation and cell migration.[16]

To study the impact of FLNA editing on cellular and organismic function, we generated mice that are specifically impaired in the editing of FLNA: the double-stranded region required for recruiting of ADAR2 is formed by an exonic and a complementary intronic region. Removal of the intronic sequence disrupts the formation of the double-stranded

Received 18 August 2022; accepted 4 November 2022;
https://doi.org/10.1016/j.omtn.2022.11.004.

**Correspondence:** Michael F. Jantsch, Medical Unversity of Vienna, Center of Anatomy and Cell Biology, Division of Cell and Developmental Biology, Schwarzspanierstrasse 17, 1090 Vienna, Austria.
**E-mail:** michael.jantsch@meduniwien.ac.at

 © 2022 The Author(s).
This is an open access article under the CC BY license (http://creativecommons.org/licenses/by/4.0/).

RNA and abolishes FLNA editing.[17] To mimic a fully edited mouse, we generated a CRISPR-induced mutation where the critical Q codon was replaced by an R codon. To reflect the single amino acid exchange in the corresponding mouse strains, we refer to them as FLNA$^R$ and FLNA$^Q$ mice, which either express fully edited FLNA$^R$ or fully unedited FLNA$^Q$. *FLNA* is an X-chromosomally expressed gene. Therefore, hemizygous males exclusively express either fully edited FLNA$^R$ or unedited FLNA$^Q$. We have shown previously that mice expressing only unedited FLNA$^Q$ display increased contraction of smooth muscle cells derived from the dorsal aorta. Moreover, mice expressing unedited FLNA$^Q$ have an elevated diastolic blood pressure and develop aortic thickening and left ventricular hypertrophy with age.[17] Importantly, samples derived from human patients with left ventricular hypertrophy also showed decreased editing of FLNA in their vascular tissue. We could thus establish a link between hypoediting of FLNA and the development of cardiovascular disease.[17]

More recently, we could also establish that *FLNA* editing affects cellular stiffness and cell migration. We could show that cells expressing edited FLNA$^R$ are stiffer and show reduced migration, while cells expressing unedited FLNA$^Q$ are softer and migrate faster in cell-migration assays.[18]

Based on these observations and based on the fact that FLNA is critically involved not only in metastasis formation but also in angiogenesis, we set out to study the impact of FLNA editing on angiogenesis with a special focus on tumor angiogenesis. We can show that hypoedited FLNA$^Q$ facilitates vascularization, thereby promoting tumor growth, while hyperedited FLNA$^R$ prevents angiogenesis and restricts tumor growth.

## RESULTS

### Characterization of constitutively edited FLNA mice

To generate mice that express constitutively edited FLNA (FLNA$^R$), the editing complementary site was deleted from intron 42 of the *Flna* gene and the CAG (Q) codon was replaced by a CGG (R) codon using a targeting vector based on the CRISPR-Cas9 approach as shown (Figure S1A). The resulting edited FLNA$^R$ mice were verified by detection of the critical codon to be CGG in cDNAs of lung tissues (Figure S1C), confirming expression of fully edited *Flna* in lungs of FLNA$^R$ mice. In parallel, the previously available unedited FLNA$^Q$ mice were tested, confirming expression of the unedited CAG coding in FLNA$^Q$ lungs (Figure S1C). Wild-type (WT) mice showed around 46% editing (Figure S1C), confirming previously reported editing levels in this tissue.[9] Next, FLNA expression was analyzed in lungs of WT, FLNA$^Q$, and FLNA$^R$ mice to test whether the altered alleles show comparable expression. Western blotting results showed that lungs did not show significant differences of FLNA expression between WT, FLNA$^Q$, and FLNA$^R$ mice (Figure S1B).

### Xenograft tumors grown in edited FLNA$^R$ hosts are much smaller and less vascularized

We showed previously that FLNA editing is highest in the mouse and human vasculature.[17] Further, mice lacking *Flna* showed clear defects in vascularization.[12] To test whether FLNA editing could also regulate neo-angiogenesis and hence tumor growth, we performed xenograft assays and subcutaneously injected B16-F10 cells into mice expressing unedited FLNA$^Q$ or fully edited FLNA$^R$. Eighteen days later, tumors were harvested and tumor weight and sizes were recorded. A striking difference was observed between the two genotypes (Figure 1A). The quantifications showed an average of 5-fold difference in tumor weight (Figure 1B) and 6-fold difference in the tumor volume (Figure 1C). Overall, when melanoma cells were grown in mouse hosts expressing fully edited FLNA$^R$, the tumor size remained dramatically smaller than tumors emerging upon injection in mice expressing unedited FLNA$^Q$. These results show that tumor growth is dramatically affected depending on the editing status of the tumor microenvironment. We also performed xenograft assays in WT and edited FLNA$^R$ mice and compared the tumor size between the two genotypes. We found that neither the tumor weight nor tumor volume was statistically different when B16-F10 cell were injected and compared between WT and edited FLNA$^R$ mice (Figures S2A and S2B).

We further analyzed the xenografted tumors to determine the extent of vascularization in tumors grown in hosts expressing edited FLNA$^R$ versus unedited FLNA$^Q$. Multiple sections from each tumor were stained with CD31 antibody, a marker of endothelial cells, and analyzed for vessel density in the tumor (Figure 1D). Since the size of the tumors of edited versus unedited mice were dramatically different, the total vessel number was normalized to the size of the tumor to calculate the vessel density. The vessel density in the tumors grown in the edited FLNA$^R$ mice was at least 2-fold lower than the one found in tumors grown in unedited FLNA$^Q$ mice (Figures 1E and 1F). These results show that *Flna* RNA editing regulates neo-vascularization in the tumor xenograft assay.

### Edited FLNA endothelial cells show reduced sprouting *ex vivo* and *in vitro*

To further check the effect of FLNA editing on angiogenesis, we tested for vascular sprouting from aortic rings. One-millimeter-thick sectioned rings from dorsal aortae of mice expressing unedited FLNA$^Q$ and edited FLNA$^R$ were placed on Matrigel in the presence of 20 ng/mL vascular endothelial growth factor (VEGF). After 72 h, the aortic rings from edited FLNA$^R$ mice showed strongly reduced outgrowth compared with outgrowth observed on aortic rings collected from unedited FLNA$^Q$ mice (Figures 2A and 2B). Quantification showed that both mean sprout length as well as a total number of sprouts were significantly reduced in aortae from mice expressing edited FLNA$^R$ compared with those from mice expressing unedited FLNA$^Q$ (Figures 2C and 2D).

To test for sprouting *in vitro*, primary endothelial cells were isolated from lungs of mice expressing unedited FLNA$^Q$ and edited FLNA$^R$ using CD31 conjugated to Dynabeads (Figure S3A) and subsequently fluorescence-activated cell sorting (FACS) sorted using ICAM2-488 to obtain a pure population of endothelial cells (Figure S3B). The FACS-sorted endothelial cells were checked for their FLNA editing levels (Figure S3C) and then used for *in vitro* 3D spheroid sprouting assay.

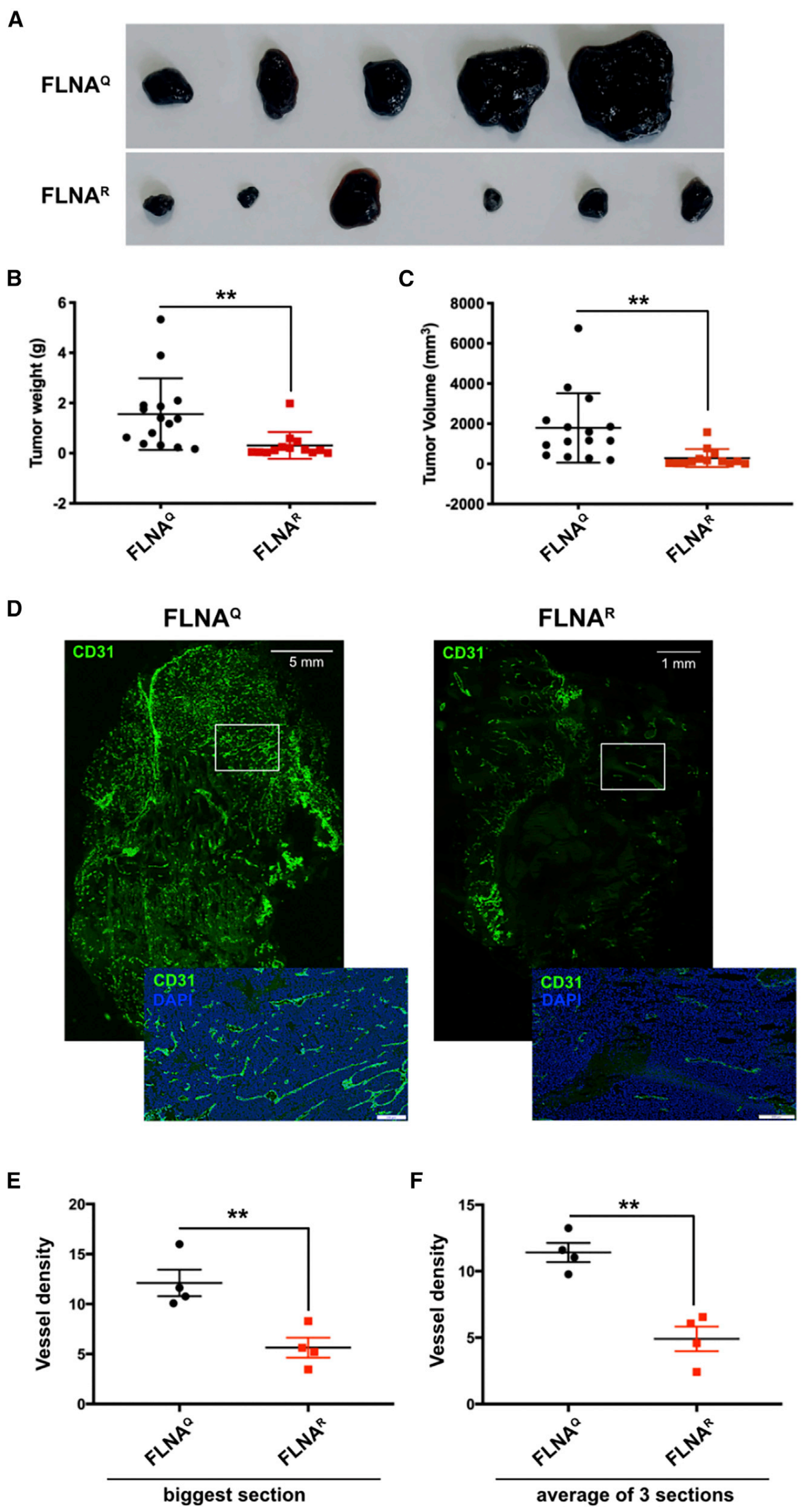


**Figure 1. Increased xenograft tumor growth in mice expressing unedited FLNA$^{Q}$ is accompanied by increased vascularization**

(A) Representative images showing xenografted tumors grown in transgenic mice expressing unedited FLNA$^{Q}$ or fully edited FLNA$^{R}$. (B and C) Graphs showing quantification and standard deviation of tumor weight (B) and tumor volume (C) grown in mice expressing unedited FLNA$^{Q}$ and constitutively edited FLNA$^{R}$. Tumor weight and volume were measured in at least 12 mice per genotype. $^{**}p < 0.01$. (D) Representative images showing immunostaining of sections of xenografted tumors using CD31 antibody (green) on tumors grown in mice expressing unedited FLNA$^{Q}$ and hyperedited FLNA$^{R}$. Insets show magnified views of the tumor sections stained with CD31 in green and counterstained with DAPI in blue. Scale bars: 5 mm and 1 mm for the low magnification sections of tumors grown in unedited FLNA$^{Q}$ and hyperedited FLNA$^{R}$ mice, respectively. High-magnification-image scale bars, 200 μm. (E and F) Graphs showing quantification and standard deviation of vessel density measured by CD31 staining compared between unedited FLNA$^{Q}$ and hyperedited FLNA$^{R}$ genotypes counted on either the biggest section (E) or by averaging of three sections from different areas of the tumor (F). The number of vessels counted in each section was normalized to the tumor area to rule out the differences due to different sample area. $^{**}p < 0.01$.

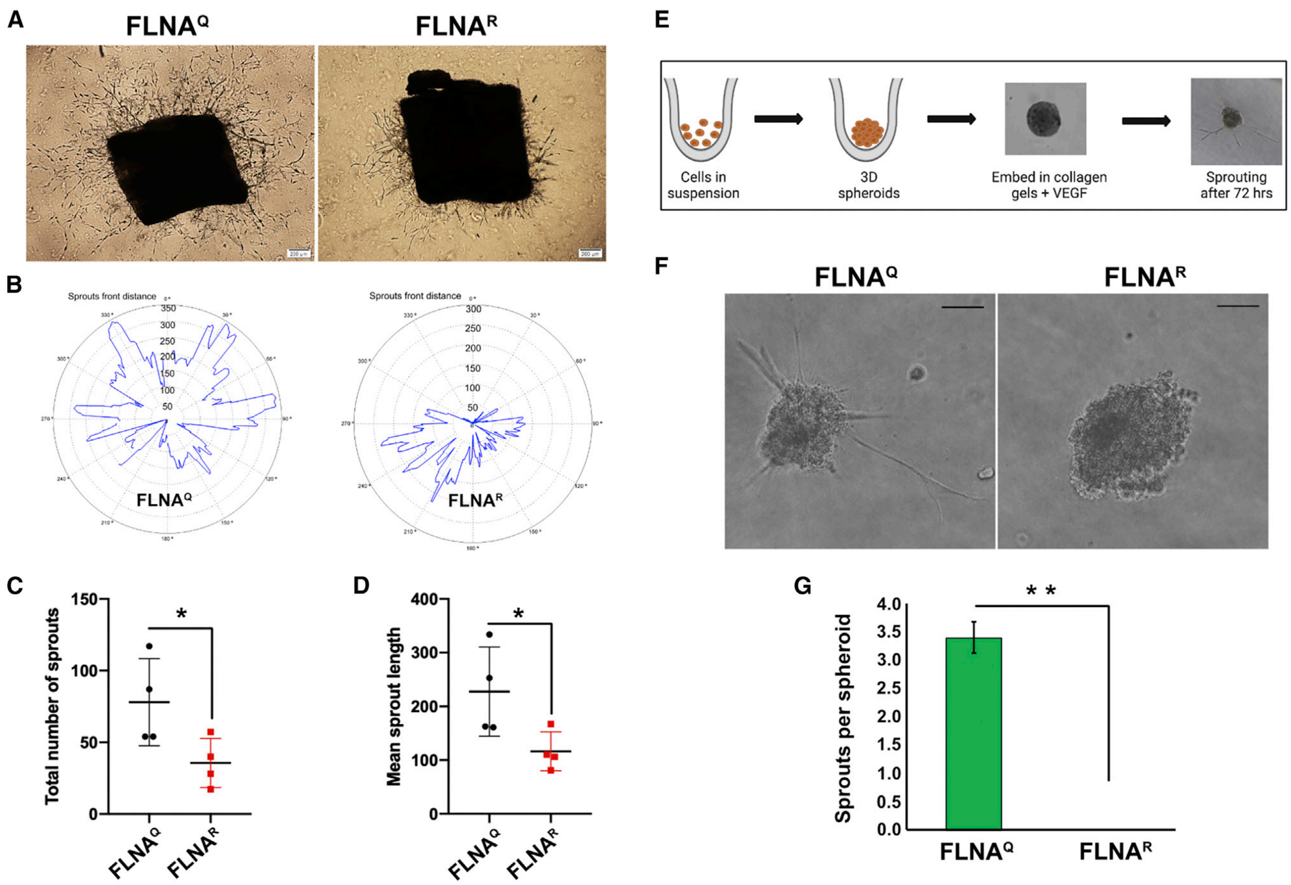


**Figure 2. Editing of FLNA reduces vascular sprouting**

(A) Brightfield images showing representative images of aortic rings taken at day 4. Aortic rings derived from mice expressing unedited FLNA$^{Q}$ show increased vascular sprouting compared with aortic rings from mice expressing edited FLNA$^{R}$. Scale bar: 200 μm. (B) Wimasis analysis of representative images showing the sprout area (blue) of aortae expressing unedited FLNA$^{Q}$ and hyperedited FLNA$^{R}$. (C and D) Graphs showing the total number of sprouts (C) and mean length of sprouts (D) compared between unedited FLNA$^{Q}$ and hyperedited FLNA$^{R}$ aortae. Four independent mice were used for the assay and at least three rings per mouse were used. *$p < 0.05$. (E) Scheme describing the steps involved in 3D spheroid generation and embedding in collagen gels for 3D sprouting assays. (F) Phase contrast images of spheroids derived from endothelial cells expressing unedited FLNA$^{Q}$ and edited FLNA$^{R}$. Sprouts were monitored after 3 days of culturing 3D endothelial spheroids. Scale bar: 50 μm. (G) Quantification of sprouts per spheroid compared between spheroids derived from mice expressing unedited FLNA$^{Q}$ and hyperedited FLNA$^{R}$.**$p < 0.01$. Data shown as mean values ±SD from three independent experiments.

The edited as well as unedited endothelial cells were cultured with methylcellulose to generate 3D spheroids (Figure 2E). The 3D sprouting assay showed that 3D spheroids derived from mice expressing fully edited FLNA$^{R}$ failed to sprout even after 72 h of collagen embedding, whereas 3D spheroids expressing unedited FLNA$^{Q}$ showed clear sprouting (Figures 2F and 2G).

### FLNA RNA editing regulates endothelial cell migration

Endothelial cell migration is an important event in formation of new vessels during both embryonic development as well as tumor angiogenesis.[19] To test for potential differences in cell migration between cells expressing unedited FLNA$^{Q}$ and edited FLNA$^{R}$, we used primary FACS-sorted endothelial cells derived from both FLNA$^{Q}$ and FLNA$^{R}$ mice and subjected them to Transwell assay. In these assays, we used VEGF as a chemoattractant. These experiments showed that endothelial cells expressing edited FLNA$^{R}$ migrate much slower toward VEGF present in the bottom chamber of the Transwell than endothelial cells expressing unedited FLNA$^{Q}$ (Figures 3A and 3B). To exclude that the differences in migration rate were not an indirect effect of different proliferation rates between endothelial cells expressing unedited FLNA$^{Q}$ and edited FLNA$^{R}$, we compared the proliferation rates using KI67 staining on CD31$^{+}$ cells. Counting of >600 CD31$^{+}$ cells in each genotype and evaluating their KI67$^{+}$ status demonstrated that the proliferation rate was not affected by the editing status of FLNA (Figures 3C and 3D). Taken together, this indicates that endothelial cells expressing edited FLNA$^{R}$ show strongly reduced migration toward a chemoattractant than do cells expressing unedited FLNA$^{Q}$.

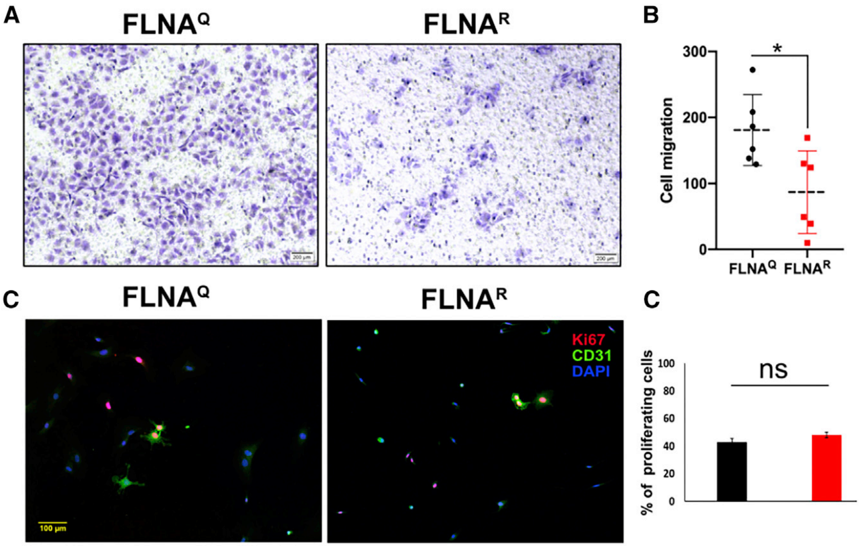


**Figure 3. Cell migration is enhanced in cells expressing unedited FLNA$^{Q}$**

(A) Images showing crystal-violet-stained endothelial cells migrating toward VEGF in a Transwell assay. Endothelial cells expressing unedited FLNA$^{Q}$ show a much-enhanced cell migration compared with hyperedited FLNA$^{R}$ cells. (B) Graph showing the quantification of cell migration between unedited FLNA$^{Q}$ and hyperedited FLNA$^{R}$ endothelial cells. Data shown are mean values ±SD from at least three independent experiments. Scale bar: 200 μm. $p < 0.05$. (C) Proliferation of endothelial cells was measured by simultaneous staining with CD31 and KI67 antibodies. (D) Cells staining positive for both antibodies were quantified and normalized over the total CD31-positive cell count.

## FLNA RNA editing regulates neo-angiogenesis in a hindlimb ischemia model

To test whether FLNA RNA editing is generally affecting neo-angiogenesis, we took advantage of a non-tumor angiogenesis model. To do so, we performed hindlimb ischemia assays on WT, FLNA$^{Q}$, and FLNA$^{R}$ mice. In this model, the femoral artery is ligated in the groin area of a mouse. The ligation forces the remaining blood flow through collateral arterioles in the adductor muscle where the increase in the shear stress and circumferential stretch of the vessel wall induces arteriogenesis (i.e., outward remodeling of pre-existing arterioles into functional arteries). Simultaneously, the lack of blood flow leads to ischemia in the lower half of the limb, which induces angiogenesis (i.e., the sprouting of new capillaries from the existing capillary bed in the adductor and gastrocnemius muscles). Briefly, the left femoral artery of mice expressing edited FLNA$^{R}$, unedited FLNA$^{Q}$, and of WT mice was ligated. The perfusion in the paw was measured shortly before surgery, immediately after surgery, as well as 3 and 7 days post ligation. Recovery rates were quite comparable at day 3 after surgery in all WT, FLNA$^{Q}$, and FLNA$^{R}$ mice (Figures 4B and S4). However, clear differences were seen 7 days post artery ligation in all three genotypes (Figure 4A). The post-ischemic blood-flow recovery expressed as the blood-flow ratio between left ligated and right non-ligated paws shows that mice expressing edited FLNA$^{R}$ show severe impairment in blood-flow recovery compared with both WT and FLNA$^{Q}$ mice (Figure 4B). On average, WT mice showed around 70% recovery at day 7 after ligation, unedited FLNA$^{Q}$ mice showed around 57% recovery, whereas edited FLNA$^{R}$ mice showed only 33% recovery at day 7 after the hindlimb ischemia surgery (Figure 4B). These results indicate that FLNA RNA editing plays an important role in post-ischemic blood-flow recovery.

Since FLNA$^{R}$ mice showed reduced blood-flow recovery post surgery, we further compared neo-angiogenesis in soleus muscles of WT, FLNA$^{Q}$, and FLNA$^{R}$ mice by immunostaining both left ligated and right non-ligated calf muscles with CD31 (Figure 4C). Comparison of CD31 intensity in the left soleus muscle showed that mice expressing edited FLNA$^{R}$ exhibited much less CD31-positive cells compared with WT and mice expressing unedited FLNA$^{Q}$ (Figure 4D), indicating that edited mice demonstrate reduced neo-angiogenesis compared with WT and mice expressing edited FLNA$^{R}$. The CD31 intensity in the right non-ischemic soleus muscle did not show any significant difference among WT, FLNA$^{Q}$, and FLNA$^{R}$ mice (Figure 4E), suggesting that no genetic difference in the steady state of vessel formation is found in any of the three genotypes used. These results suggest that FLNA RNA editing controls neo-angiogenesis during the post-ischemic blood-flow recovery.

Arteriogenesis is an important phenomenon during post-ischemic formation of new vessels.[20] To evaluate arteriogenesis in the hindlimb ischemia model, the adductor muscles of WT mice and mice expressing unedited FLNA$^{Q}$ and edited FLNA$^{R}$ were stained with an antibody directed against smooth muscle actin (SMA) (Figure 4F). Both the lumen diameter and the number of the collaterals were determined. The SMA staining showed that neither the number of collaterals (Figure 4G) nor the lumen measured by the diameter of the collaterals (Figure 4H) was significantly changed among WT, FLNA$^{Q}$, and FLNA$^{R}$ mice 7 days post ischemia. Overall, these results suggest that the post-ischemic reduced blood flow in mice expressing edited FLNA$^{R}$ is due to defects in angiogenesis and not due to defective arteriogenesis.

## FLNA RNA editing controls VEGFR2 signaling in endothelial cells

During neo-angiogenesis, endothelial cell migration and proliferation are the two most important cellular events playing a vital role in formation of new vessels in response to VEGF. VEGFR2 (*Kdr*) is the key receptor regulating VEGF-mediated endothelial cellular effects.[20] Upon binding of VEGF-A ligand to VEGFR2, the receptor undergoes conformational changes and leads to activation of several downstream events, including focal adhesion kinase (FAK) activation that mediates endothelial cell migration.[21] We checked the phosphorylation of FAK (pFAK) at Tyr397, which is important for focal adhesion-mediated adhesion and migration by staining endothelial cells expressing edited FLNA$^{R}$ or

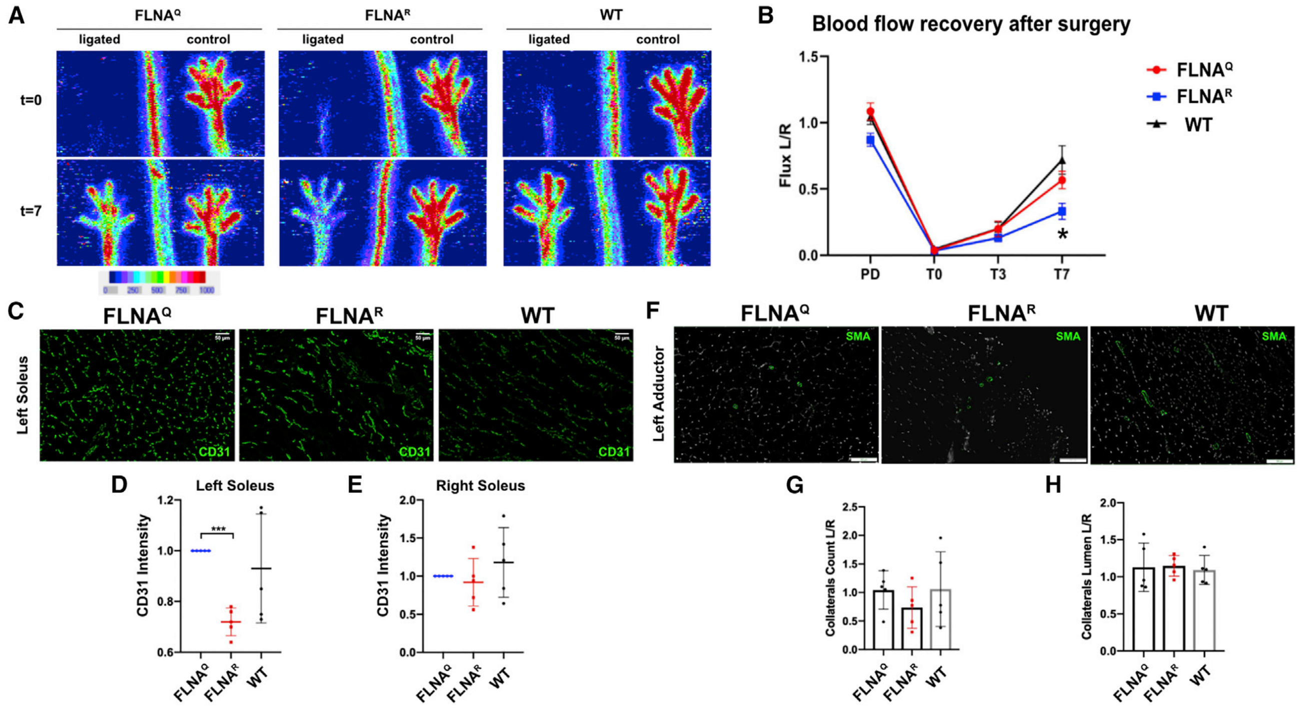


**Figure 4. Mice expressing edited FLNA$^R$ show delayed post-ischemic recovery of blood flow**

(A) The left femoral artery of mice of different genotypes was ligated and post-ischemic blood flow was measured using LDPI. Representative images showing the left and right (control) paw compared among unedited FLNA$^Q$, hyperedited FLNA$^R$, and WT mice. Paw perfusion was measured both before ligation (t = 0) and then after 7 days of surgery (t = 7). Ten mice per genotype were used for the hindlimb ischemia assay. (B) Graph showing the post-ischemic blood-flow recovery measured as a flux ratio of left (ischemic) versus right (control) paw perfusion in unedited FLNA$^Q$, hyperedited FLNA$^R$, and WT mice. Data are presented as mean ±SEM. *$p < 0.05$. (C) Representative images showing left ligated soleus muscle stained with CD31 in green compared among unedited FLNA$^Q$, hyperedited FLNA$^R$, and WT mice. Scale bar: 50 μm. (D and E) Graphs showing the quantification of CD31 intensity in left ligated (D) and right unligated (E) soleus muscles. ***$p < 0.01$. (F) Representative images showing left ligated adductor muscle stained with smooth muscle actin (SMA) in green compared among unedited FLNA$^Q$, hyperedited FLNA$^R$, and WT mice. Scale bar: 100 μm. (G and H) Graphs showing the quantification of number of collaterals (G) and lumen of collaterals (H) represented as a ratio of left/right adductor muscle. Data are presented as mean ±SEM. No significant difference is observed between either the count or lumen of collaterals in the adductor muscle.

unedited FLNA$^Q$ that were treated with or without VEGF (Figure 5A).[22] We found that pFAK staining at Tyr397 is significantly increased in FLNA$^Q$ endothelial cells compared with FLNA$^R$ cells when these cells were stimulated with VEGF for 30 min (Figure 5B). However, not much difference in pFAK levels was observed between the untreated endothelial cells expressing unedited FLNA$^Q$ and edited FLNA$^R$, indicating that the steady-state levels of VEGFR2 signaling are not affected. We also checked the steady-state levels of several VEGF-receptors, such as VEGFR1, VEGFR2, and VEGFR3, along with integrin β3 by qPCR analysis from the cDNA of untreated endothelial cells expressing unedited FLNA$^Q$ and edited FLNA$^R$ and found that all these membrane receptors are equally expressed in both cell types (Figure S5A).

Upon VEGF ligand binding, VEGFR2 activation also promotes its own internalization. The internalized receptor is either subjected to lysosomal mediated degradation or is recycled back to the membrane.[21] We further compared the fate of the internalized VEGFR2 in endothelial cells expressing unedited FLNA$^Q$ and edited FLNA$^R$ by treating them with VEGF and then immunostaining them with total VEGFR2 antibody (Figure 5C). We observed that, after stimulating cells with VEGF for 30 min, VEGFR2 was dramatically reduced in endothelial cells expressing edited FLNA$^R$, whereas VEGFR2 seemed to be protected from degradation in endothelial cells expressing unedited FLNA$^Q$ (Figures 5C and 5D). The steady-state levels of VEGFR2 in the absence of VEGF were found to be similar (Figure 5D). Taken together, this suggests that VEGFR2 has a higher turnover rate in cells expressing edited FLNA$^R$.

We also checked the membrane VEGFR2 expression directly on tumor sections from xenografts in mice expressing unedited FLNA$^Q$ and edited FLNA$^R$ by immunostaining the tumor sections with extracellular domain (ECD)-specific VEGFR2 antibody. Our results showed that membrane VEGFR2 receptor expression was seemingly unchanged between the tumor xenografts in either FLNA$^Q$ or FLNA$^R$ host mice (Figure S5B).

## FLNA editing status of melanoma cells slows down liver metastasis

So far, we have investigated how the editing status of the vasculature affects xenograft tumor growth and neovascularization upon

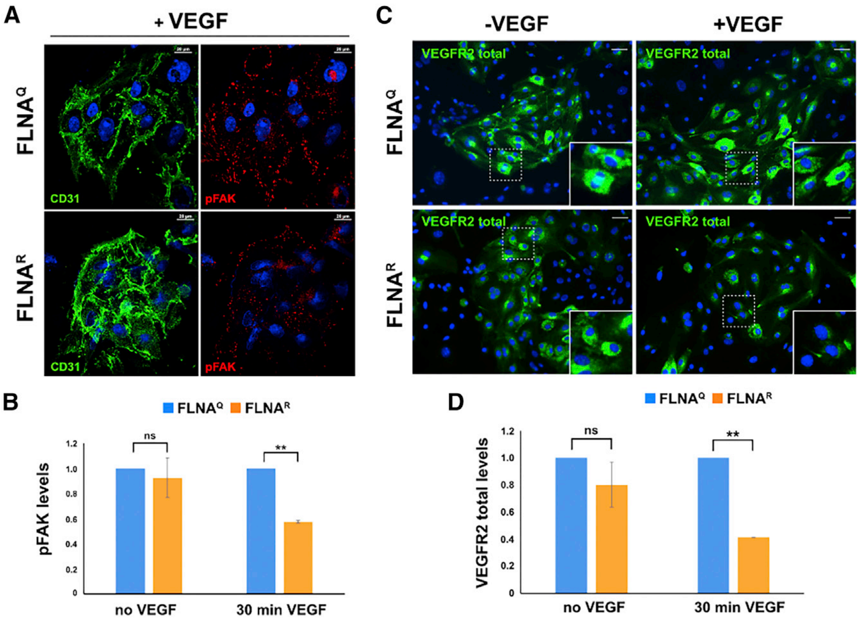


**Figure 5. VEGFR signaling is reduced in cells expressing edited FLNA$^R$**

(A) To test whether VEGFR signaling is altered in endothelial cell expressing unedited FLNA$^Q$ or edited FLNA$^R$, we stained lung endothelial cells that were CD31 positive with an antibody against phosphorylated FAK (pFAK) (scale bar: 20 μm). (B) Quantification of >150 cells in three independent experiments revealed that FAK phosphorylation was significantly reduced in cells expressing hyperedited FLNA$^R$ upon VEGF stimulation. (C) Staining for VEGFR2 also showed a clear reduction of this receptor in cells expressing edited FLNA$^R$ upon stimulation with VEGF (scale bar: 100 μm). (D) Quantification of VEGFR2 staining on >250 cells. Data are presented as mean ±SD

induced hindlimb ischemia. Next, we investigated how the *Flna* RNA editing status of tumors affects their growth and metastatic potential. To do so, we manipulated B16 melanoma cells to express either constitutively edited FLNA$^R$ or the unedited FLNA$^Q$ version. This was achieved using CRISPR-Cas9-mediated mutagenesis as shown in Figure S6A. The repair template deleted the editing complementary sequence to prevent editing and introduced a genomic arginine codon to generate cells expressing edited FLNA$^R$. An intronic GFP reporter was used to sort GFP-positive single cells. Those were expanded to clones and screened using PCR (Figure S6B). PCR-positive clones were confirmed by sequencing and their FLNA editing was confirmed at both genomic as well as cDNA levels. Clones that showed complete CAG (unedited) or CGG (edited) sequences in their cDNA amplicons were selected (Figure S6C). The final edited and unedited B16-F10 clones were checked for their FLNA expression by western blotting, and we found that these clones had a similar but reduced expression of FLNA protein compared with WT cells (Figure S6D). The reduced expression may be a side effect of the introduced GFP reporter.

WT and the CRISPR-modified B16 melanoma cells expressing unedited FLNA$^Q$ or edited FLNA$^R$ were injected subcutaneously in WT mice to assess the autonomous role of FLNA RNA editing present in tumor cells on tumor growth *in vivo* (Figure S7A). The xenograft assay showed no significant difference in tumor growth with respect to tumor weight (Figure S7B) or tumor volume (Figure S7C). In order to assess the autonomous effects of FLNA editing on organ-specific metastasis, we performed tail-vein injections of WT B16-F10 cells and cells expressing unedited FLNA$^Q$ or edited FLNA$^R$ in WT C57Bl6 mice. After 14 days, mice were sacrificed and their lungs and livers were analyzed for the occurrence of metastases. Microscopic examination of lung tissues did not show any difference in the number of metastatic nodules (Figure 6C). However, mice injected with melanoma cells expressing pre-edited FLNA$^R$ showed reduced liver metastasis compared with mice injected with WT melanoma cells or cells expressing unedited FLNA$^Q$ (Figure 6A and 7B). Liver metastasis was observed by bleaching the tissue with Fekete's solution and then counting metastatic nodule (black) microscopically with sample genotype not being revealed.

## DISCUSSION

Filamins are critical actin-crosslinking proteins that link the cortical cytoskeleton to the cell surface. In fact, filamin A has been shown to interact with integrins.[23] Consequently, filamin expression has been shown to affect metastasis formation.[14] In this study, we investigate the impact of filamin RNA editing on angiogenesis. Filamin A is edited in Ig-repeat 22, close to the region mapped to interact with integrins. Moreover, repeat 22 has been shown to be critically involved in the interaction with beta-arrestin.[24]

We can show that mice expressing constitutively edited FLNA$^R$ show reduced angiogenesis *in vitro* as well as *in vivo*. Using a xenograft tumor assay, we can show that reduced angiogenesis correlates with reduced tumor growth. Conversely, using a hindlimb ischemia assay, mice only expressing hyperedited FLNA$^R$ show delayed recovery and reduced perfusion of the ischemic paw. In contrast, mice lacking filamin A editing and therefore only expressing FLNA$^Q$ show increased xenograft tumor growth and have increased perfusion following induced hindlimb ischemia. These findings are consistent with analysis of The Cancer Genome Atlas (TCGA) data that indicate reduced filamin A editing in tumor versus healthy tissues.[25] Thus, decreased editing is most likely facilitating tumor vascularization. Reduced filamin A editing can also be beneficial for post-ischemic neo-angiogenesis.

Molecularly, the reduced angiogenesis in mice with increased filamin A editing is most likely linked to a decrease in VEGFR2 signaling observed in FLNA$^R$-expressing endothelial cells as suggested by our

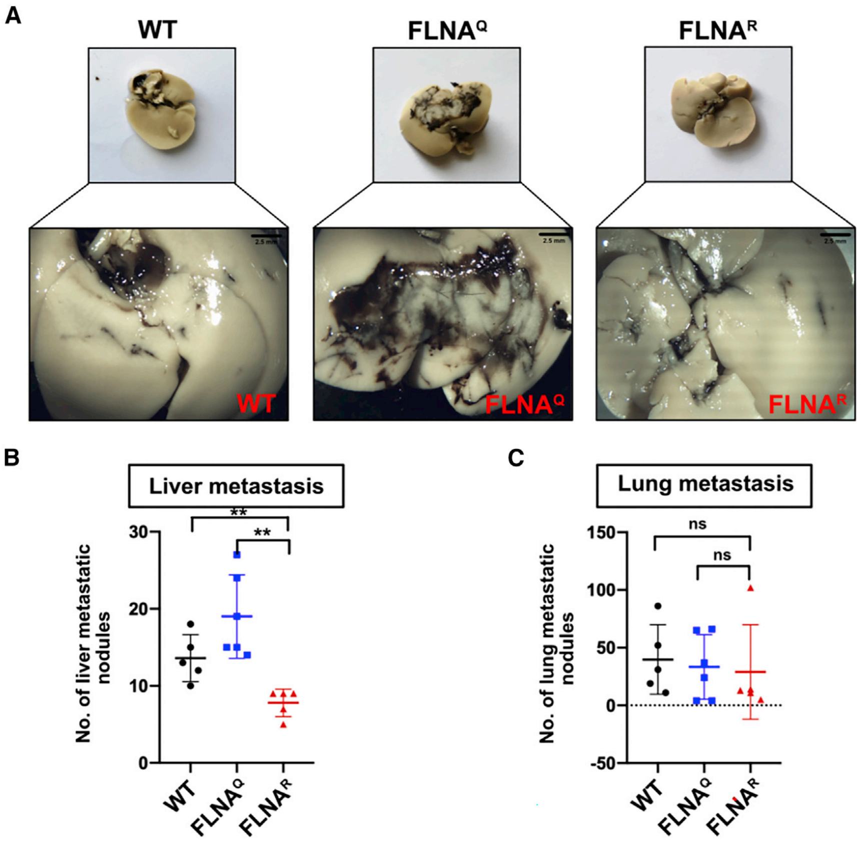


**Figure 6. The FLNA editing status of B16 melanoma cells affects their metastatic potential**

(A) Representative images of livers of WT mice after tail-vein injection of WT, unedited $FLNA^Q$, and hyperedited $FLNA^R$ B16 CRISPR clones. Insets show the respective magnified view of the part of liver of all the three genotypes. The black area indicates the metastatic tissue. Scale bar: 2.5mm. (B) Graph showing the quantification of liver metastasis represented by number of metastatic nodules in livers injected with WT, unedited $FLNA^Q$, and hyperedited $FLNA^R$ CRISPR clones. (C) Graph showing the quantification of lung metastasis represented by number of metastatic nodules in lungs injected with WT, unedited $FLNA^Q$, and hyperedited $FLNA^R$ CRISPR clones. Graph indicates mean and 75 percentile. $^{**}p < 0.01$; ns, non-significant.

experiments (Figure 7). Reduced levels of VEGFR2 upon VEGF stimulation of cells expressing edited FLNARs are most likely the result of altered receptor turnover. While we have attempted to directly test for receptor turnover, these experiments were not conclusive. FLNA crosslinks the cortical actin cytoskeleton and also interacts with transmembrane proteins such as integrins.[26] Altered membrane dynamics in response to FLNA editing status might therefore occur and explain the phenomena observed here.

This, in turn, suggests that editing of filamin A might be regulated dynamically *in vivo* to allow adaptation to altered extra- and intracellular needs. In fact, this is in good agreement with our observation that editing of filamin A and filamin B pre-mRNAs do not strictly correlate: both pre-mRNAs are edited at the same exon, leading to the same amino acid exchange in FLNA and FLNB.[9,27] However, filamin A editing is highest in the dorsal aorta and the colon, where editing can reach almost 100%. In contrast, editing of filamin B is modest in the vasculature and colon but can reach levels of 80% in cartilage and brown fat.[27] Editing is also regulated during development. For instance, editing of filamin A is negligible during embryogenesis and increases postnatally to reach a plateau at ~1 month of age. This finding would be consistent with increased vascularization occurring during embryonic and post-natal development, while vascularization would decrease correlating with an increase in filamin A editing later in development.

Interestingly, we can also show that the editing status of tumors can also affect metastasis formation in a tissue-specific manner. Tail-vein-injected B16 melanoma cells that were modified using CRISPR-Cas9 to express only edited or unedited FLNA showed comparable tumor formation in the lungs. However, melanoma cells expressing unedited $FLNA^Q$ showed much higher metastasis formation in the liver than WT B16 cells or cells expressing edited $FLNA^R$. At present, it is not clear why B16 cells expressing unedited $FLNA^Q$ show increased tumor formation in the liver. However, it is possible that integrin-mediated cell adhesion alters the preference of the modified B16 cells for different tissue environments. Alternatively, we could show that cells expressing $FLNA^Q$ are softer, while cells expressing pre-edited $FLNA^R$ are more rigid.[17] It may therefore be possible that the different preferences for target organs is affected by the migratory and/or adhesive potential of the modified B16 cells.

In summary, our study shows that editing of filamin A pre-mRNA can be friend of foe, depending on the condition and type of cell investigated. Filamins are edited largely by ADAR2. Editing of filamin A is regulated throughout development and shows tissue-specific differences. Given the fact that FLNA editing can be both beneficial and detrimental, it is obvious that FLNA editing needs to be dynamically regulated. We have shown previously that editing levels can be regulated by the rate of RNA processing.[28] Moreover, the availability of ADAR2 and its access to the editing region will be a strong regulator of filamin A editing. Given the high disease relevance of FLNA editing, it will be interesting to determine how FLNA editing is regulated in human disease conditions other than tumor formation. Moreover, FLNA editing may also be exploited therapeutically to promote or repress angiogenesis or metastatic potential.

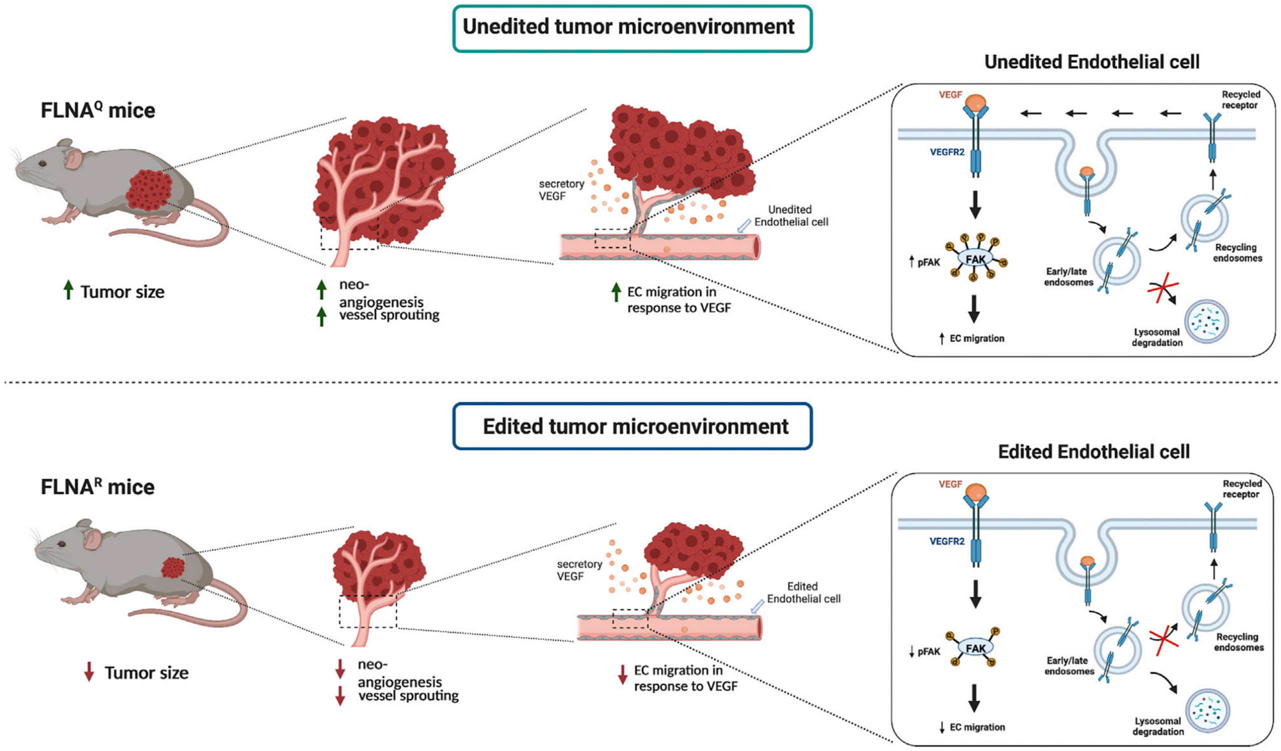


**Figure 7. Model of altered angiogenesis in mice expressing edited or unedited FLNA**

Mice expressing unedited FLNA$^{Q}$ display increased angiogenesis compared with mice expressing hyperedited FLNA$^{R}$. Consequently, xenografted tumors grow larger in FLNA$^{Q}$ mice. (Right) Endothelial cells expressing unedited FLNA$^{Q}$ show elevated response to VEGF, most likely as a consequence of reduced VEGFR2 receptor turnover.

## MATERIALS AND METHODS

### Generation of constitutively edited FLNA transgenic mice

Mice expressing constitutively edited FLNA$^{R}$ were generated in collaboration with Cyagen (Santa Clara, CA, USA) using CRISPR-Cas9. A targeting vector was designed to delete a 228-bp-long region in intron 42–43 harboring the editing complementary site that is required for *Flna* editing and with a point mutation to convert CAG to CGG to create a constitutively edited version of FLNA. Briefly, mouse genomic fragments containing the homology arms were amplified from bacterial artificial chromosome (BAC) clone using high-fidelity Taq and were sequentially assembled into a targeting vector together with recombination sites. The schematic representing the donor vector along with the targeted allele is shown in Figure S1. The donor DNA containing Q1682R (CAG to CGG) and Cas9 mRNA were co-injected into fertilized mouse eggs to generate targeted knockin offspring. F0 founder animals were identified by PCR followed by sequence analysis. The positive founders were then bred with WT mice to test germline transmission and F1 animal generation. Transgenic mice were backcrossed with C57BL/6J for six generations to generate isogenic lines. Genotyping was performed by PCR on DNA isolated from finger biopsies using primers forward, TCTGGATGGTAGGCTTCTGC, and reverse, CTGGAAGCATAGC AGATGTGG. Forward primer is located in exon 42 while the reverse primer is located in intron 42 downstream of the editing complementary sequence. In WT animals, the primer pair produces a band of 520 bp; in unedited FLNA$^{Q}$ animals, a band of 417 bp is produced. Pre-edited FLNA$^{R}$ mice show a band of 385 bp due to the lack of a *loxP* site in the CRISPR repair construct. All animal experiments were conducted in accordance with national regulations.

### Western blotting of mouse tissue lysates

Lung tissues were also lysed with radioimmunoprecipitation assay (RIPA) buffer and homogenized using a Dounce homogenizer. The samples were then centrifuged at 15,000 rpm for 30 min at 4°C and the supernatant was mixed with 2× SDS sample buffer, boiled, and loaded on SDS-PAGE gels. Lung lysates were probed with a monoclonal FLNA antibody 1B6.[18] GAPDH was used as a loading control. All blots were detected by chemiluminescence and images using a CCD camera on a fusion-FX (Fisher Biotec, West Perth, WA, Australia). All experiments were done at least in triplicate and mean values ±SD were plotted. Statistical significance was calculated using Student's t test.

### cDNA preparation and determination of FLNA editing levels

Total RNA was isolated from homogenized organs with TRIzol reagent. After DNAse I treatment, cDNAs were synthesized using M-MLV Reverse Transcriptase kit (Invitrogen, Carlsbad, CA) and random hexamer primers. An FLNA cDNA fragment was amplified using primers spanning the spliced editing regions exon 42 to exon 43 of FLNA. Amplified products were gel eluted and sent for

sequencing to check editing levels. For amplification, forward primer was 5′ GTCAAGTTCAACGAGGAGCAC-3′ and reverse primer was 5′ GTGCACCTTGGCATCAATTGC-3′.

For qPCR assays, two different sets of primers were used to amplify pre-mRNA and mRNA from various mouse organ tissues. Each tissue was processed in biological triplicates, including no-template control (ntc) and room-temperature (RT) samples as control. Primers used are discussed next.

#### Pre-mRNA

MJ6905: forward primer, CTGATAGCCCCTTCGTGGTG.

MJ6976: reverse primer, TTTCTGCGTAGGCACTCAGC.

#### mRNA

MJ6905: forward primer, CTGATAGCCCCTTCGTGGTG.

MJ6974: reverse primer, CCCCTTGGCTCCATTCAGAC.

#### Tumor xenograft assay

WT B16-F10 cells were cultured to 50% confluency and harvested. Cells were washed once with PBS and then resuspended in ice-cold Hank's balanced salt solution (HBSS). The cell suspension was passed through a 70-μm cell strainer and counted using trypan blue. Viability was verified to be >90%. Cell concentration was adjusted to $1 \times 10^6$ cells/mL using ice-cold HBSS. Cells were injected using a 1-mL syringe and a 27G needle. One-hundred microliters of cell suspension was injected subcutaneously per mouse. Tumors were harvested 17 days after injection, and 8- to 12-week-old WT, $FLNA^Q$, and $FLNA^R$ mice were used for the assay. The weight (grams) and the volume of the tumor (cubic millimeters) were measured and imaged. The volume of the tumor was calculated as (length $\times$ width $\times$ height)/2. The tumor samples were further processed for cryo-sectioning.

#### Immunostaining of tumor sections

The cryosections were fixed with ice-cold methanol for 15 min at $-20^\circ$C and with ice-cold acetone for 5 min at $-20^\circ$C. The sections were then blocked with 5% BSA for 30 min at RT. To stain for vessels, a CD31 antibody (BD Biosciences), a cell surface marker for endothelial cells was used along with secondary $\alpha$-Rat Alexa 488. Slides were then washed and mounted using antifade containing DAPI. The tumor sections were imaged using VS120-L100-W Olympus slide scanner. The images were analyzed using ImageJ and total number of vessels was counted per sample. The total number of vessels was normalized to the sample area and plotted. Multiple sections from each sample were analyzed and averaged.

#### Aortic ring sprouting assay

Thoracic aortae were dissected from 8-week-old $FLNA^Q$ and $FLNA^R$ mice. Aortae were cleaned of surrounding fat and cut into 1-mm rings using a stereo-microscope and a scale ruler. Three rings were used per mouse for each experiment. Forty-eight-well plates (Corning) were coated with 100 μL of Matrigel per well and kept at 37°C for 30 min. Aortic rings were placed on the Matrigel and incubated at 37°C for 5 min. Subsequently, 50 μL of Matrigel was overlayed on rings and placed at 37°C, again for 40 min, and 250 μL of medium was placed on top, containing 20 ng/mL VEGF (Peprotech). Rings were incubated at 37°C in a $CO_2$ incubator. After 4 days, images were taken. Aortic ring sprouting was quantified using an online microscopy analysis platform (wimasis.com). The identity of the samples submitted to Wimasis was not revealed and was blinded.

#### Primary endothelial cell isolation

Lungs from 5- to 6-week-old mice were isolated and homogenized using collagenase type I (Sigma). The resulting cell suspension was resuspended using an 18G needle, filtered through a 70-μm cell strainer, and pelleted. The cell pellets were resuspended and incubated with CD31 antibody (BD Biosciences)-coupled Protein G Dynabeads (Thermo Scientific) for 1 h at 4°C. The beads were separated in a magnetic separator, washed three times with medium, and cells were plated on fibronectin-coated dishes. Medium was exchanged every 48 h until cells were confluent and subsequently used for FACS using ICAM2-488 (Thermo Scientific) to enrich for endothelial cells. After FACS, cells were directly used for the experiments.

#### 3D spheroid sprouting assay

After FACS, 2,000 endothelial cells were plated in a non-coated, round (U)-bottom 96-well dish along with 5-mg/mL methylcellulose to generate 3D spheroids. After 24 h, the 3D spheroids were pelleted and embedded in a collagen bed using collagen type I (Gibco). Pictures of the sprouts were taken after 72 h.

#### Transwell migration assay

Eight-micrometer, 24-well Transwell inserts (Corning) were coated with 10 μg/mL fibronectin for 1 h at 37°C. Then 50,000 endothelial cells of each genotype were resuspended in DMEM + 0.5% FBS and put in the upper chamber of the Transwell insert. In the bottom chamber, 600 μL of medium containing DMEM + 0.5% FBS + VEGF (25 ng/mL) was placed. The Transwell inserts were incubated for 16 h at 37°C. After 16 h, the non-migrated cells from the top chamber were removed using a wet cotton swab and the migrated cells on the lower side of the membrane were stained with crystal violet and counted under a microscope.

#### Proliferation assay

Twenty-thousand primary endothelial cells were seeded onto fibronectin-coated coverslips for Ki-67 staining. After fixation with 4% PFA for 10 min, cells were permeabilized with 0.1% Triton X-100 for 15 min. Unspecific binding sites were blocked by incubating cells in 2% BSA for 1 h, followed by the antibody staining with Ki-67 recombinant rabbit monoclonal antibody (1:100; Invitrogen; #MA5-14520) and purified rat anti-mouse CD31 antibody (1:100; BD biosciences; #557355) overnight at 4°C. Incubation with secondary anti-rabbit Alexa 647 and anti-mouse Alexa 488 was performed for 1.5 h at RT. The cells were mounted with antifade containing DAPI. Antibody staining and DAPI were visualized with the

Olympus slide scanner BX61VS and the VS-ASW-S6 software. Only CD31-positive endothelial cells were included in the following analysis. Ki-67- and DAPI-positive nuclei were counted from ~200 CD31-positive cells and the DAPI/Ki-67 ratio calculated. The statistical analysis was performed with SPSS and the independent t test (n = 2).

#### Hindlimb ischemia assay

These experiments were approved by the Committee on Animal Welfare of the Leiden University Medical Center (Leiden, the Netherlands) and were performed in accordance with Directive 2010/63/EU of the European Parliament and Dutch government guidelines, and 14- to 15-week-old male WT C57Bl6/J, $FLNA^{Q}$, and $FLNA^{R}$ mice were used for the assay. The experiment was performed following the same protocol as described before. Briefly, mice were anesthetized via intraperitoneal injection of midazolam (5 mg/kg; Roche Diagnostics, Almere, the Netherlands), medetomidine (0.5 mg/kg; Orion, Espoo, Finland), and fentanyl (0.05 mg/kg; Janssen Pharmaceuticals, Beerse, Belgium). Unilateral hindlimb ischemia (HLI) was induced by double ligation of the left femoral artery, proximal to the superficial epigastric artery and proximal to the bifurcation of the popliteal and saphenous artery. After surgery, mice were given a subcutaneous injection of flumazenil (0.5 mg/kg, Fresenius Kabi, Utrecht, the Netherlands) and atipamezol (2.5 mg/kg, Orion) to antagonize anesthesia. Buprenorphine (0.1 mg/kg, MSD Animal Health, Boxmeer, the Netherlands) was given after surgery for pain relief.[29,30]

Blood-flow recovery was measured using laser Doppler perfusion imaging (LDPI). The measurements were taken pre-surgery, immediately after surgery, and then at day 3 and day 7 in both the left (ischemic) and the right (non-ischemic) paw. Before measurements, mice were anesthetized with an intraperitoneal injection of midazolam (5 mg/kg, Roche Diagnostics) and medetomidine (0.5 mg/kg, Orion). Mice were placed in a double-glassed pot that was perfused with water at 37°C for 5 min prior to each measurement. LDPI measurements in the ligated paw were normalized to measurements of the unligated paw as an internal control. After LDPI, anesthesia was antagonized by subcutaneous injection of flumazenil (0.5 mg/kg) and atipamezole (2.5 mg/kg).

At day 10 after surgery, mice were sacrificed and gastrocnemius, soleus, and adductor muscles were harvested for immunohistochemical analysis. Adductor muscles were fixed with 4% paraformaldehyde and embedded in paraffin. Soleus and gastrocnemius muscles were snap frozen and stored at −80°C until sectioned.

#### Immunostaining on hind-limb-ischemic muscles

Six-micrometer cryosections of the soleus muscle were taken and stained with CD31 (BD Biosciences) antibody to visualize endothelial cells. The stained samples were imaged using a slide scanner (BX61VS, Olympus) and vessel density was analyzed by measuring CD31 intensity per sample using ImageJ (Fiji) and normalized to the total sample area. Five-micrometer paraffin sections of the adductor muscle were taken and stained with SMA antibody (SIGMA, St. Louis, MI) to detect smooth muscle cells. The stained samples were imaged using a slide scanner (BX61VS, Olympus) and analyzed. Three sections of each sample were analyzed and averaged. The smallest diameter of each visible lumen was measured and the total number of arterioles per section/area was counted.

#### Endothelial cell assays

For cell signaling assays, primary endothelial cells were isolated using CD146 (LSEC)-coupled microbeads (Miltenyi Biotec). Briefly, lungs were harvested from $FLNA^{Q}$ and $FLNA^{R}$ mice, collagenase treated, and then incubated with CD146 microbeads. Labeled cell suspension was then subjected to magnetic cell sorting (MACS) purification and the eluents were plated on fibronectin-coated dishes. For pFAK studies, double staining with pFAK (Invitrogen) and CD31 (BD Biosciences) was performed to identify endothelial cells in both $FLNA^{Q}$ and $FLNA^{R}$ at steady state and after treating cells with VEGF (25 ng/mL) for 30 min. The pFAK intensity was measured by total cell fluorescence intensity using ImageJ, and the average pFAK intensity per cell was plotted and compared between $FLNA^{Q}$ and $FLNA^{R}$ genotypes in the presence and absence of VEGF (Peprotech). In order to check the VEGFR2 receptor stability after VEGF stimulation, we treated both $FLNA^{Q}$ and $FLNA^{R}$ endothelial cells with VEGF (25 ng/mL) for 30 min and stained with total VEGFR2 antibody (Cell Signaling Technology, Leiden, the Netherlands). The total VEGFR2 signal was then analyzed by measuring total cell fluorescence using ImageJ. The internalized VEGFR2 receptor levels per cell was calculated and compared between $FLNA^{Q}$ and $FLNA^{R}$ genotypes in the presence and absence of VEGF. In order to specifically look at the VEGFR2 receptor present at the membrane, we stained endothelial cells with an ECD-specific VEGFR2 antibody (R&D Systems) at the steady state and after treating the cells with VEGF (25 ng/mL) for 30 min. The membrane VEGFR2 receptor intensity per cell was plotted and compared between $FLNA^{Q}$ and $FLNA^{R}$ genotypes in the presence and absence of VEGF. All the endothelial cell assays were done in triplicate, mostly with three different biological primary endothelial cell isolates. For every replicate, at least 50–60 cells per genotype per condition were analyzed to measure the intensity.

#### CRISPR-Cas9 in B16-F10 melanomas

To generate edited and unedited B16 melanoma cells, the same guide RNAs (gRNAs) were used as for the generation of constitutively edited transgenic mice. gRNA1 (CTGTTTCTAGTCTTCAGGTG) targeting exon 42 and gRNA2 (GCTACCTGGGTGTGTGATTG) targeting intron 42 were cloned in vector p31 containing T7 promoter followed by BbsI cloning sites. *In vitro* gRNA transcription was performed with HiScribe T7 High Yield RNA synthesis kit (NEB) according to the manufacturer's protocol and gRNA was purified via phenol:chloroform:isoamyl alcohol (25:24:1, Applichem, A2279) followed by ethanol precipitation. WT B16-F10 cells were electroporated with 12 μg of gRNA pre-mixed with 5 μg of recombinant Cas9 protein in Cas9 buffer (20 mM HEPES pH 7.5, 150 mM KCl, 0.5 mM DTT, 0.1 mM EDTA) using a Neon transfection system. After 48 h of electroporation, batch culture was collected for genotyping to confirm editing. Editing efficiency was analyzed with TIDE algorithm

(https://tide.deskgen.com/) based on chromatogram analysis with WT PCR product used as a reference.

A repair template was generated specific to either unedited (CAG) or edited (CGG) comprising left and right homology arms spanning the editing site and editing complementary site along with a GFP marker in intron 43. WT B16-F10 cells were electroporated with 12 μg of gRNA each pre-mixed with 5 μg of Cas9 protein in Cas9 buffer and 7 μg of the repair template. The batch culture was genotyped after 48 h, and single cells were then sorted in 96-well plates using GFP as a marker. The single cell clones were expanded and screened first using PCR. The PCR-positive clones were then fully sequenced from left homology arm to right homology arm. The clones that showed the desired sequence were next tested for FLNA editing by Sanger sequencing and FLNA expression by western blotting.

### Tail-vein assay

WT, $FLNA^Q$, and $FLNA^R$ B16-F10 cells were kept in log phase until harvested. The tail veins of mice were dilated and 250,000 cells were injected per mouse using 27G 0.5-inch needle as described previously.[31] After 14 days, mouse lungs and livers were harvested, rinsed with cold PBS, and imaged. Both the organs were counted for metastatic nodules after bleaching with Fekete's solution (95% ethanol, 37% formaldehyde, glacial acetic acid). Metastatic nodules (black) were counted microscopically with sample genotype not being revealed. Either 5 or 6 mice were used per genotype.

### Statistical analysis

Data were analyzed using GraphPad Prism or Microsoft Excel. Statistical analysis was done using Student's t test with equal variance, and $p < 0.05$ was considered to be statistically significant.

### Data availability

No genomic data were collected in this study.

## SUPPLEMENTAL INFORMATION

Supplemental information can be found online at https://doi.org/10.1016/j.omtn.2022.11.004.

## ACKNOWLEDGMENTS

CRISPR-modified B16 cells were generated by the Vienna Biocenter Core Facilities genomics division. Figures were generated using BioRender. We thank Robert Vilvoi and Stephan Hemm for animal handling. Marcel Kertesz is thanked for mouse genotyping. This work was supported by the Austrian Science Fund, grant numbers P 32678 and F80-07 to M.F.J.

## AUTHOR CONTRIBUTIONS

M.J., A.Y.N., and M.F.J. conceptualized the study and wrote the manuscript. M.J., G.M., K.M., M.R.d.V., M.J., and L.K. performed experiments. M.F.J. finalized the manuscript and supervised the work.

## DECLARATION OF INTERESTS

M.F.J. and the Medical University of Vienna have filed a European patent for the therapeutic use of FLNA editing to control tumor growth, inflammation, and angiogenesis.

## REFERENCES

1. Nishikura, K. (2016). A-to-I editing of coding and non-coding RNAs by ADARs. Nat. Rev. Mol. Cell Biol. *17*, 83–96. https://doi.org/10.1038/nrm.2015.4.
2. Samuel, C.E. (2019). Adenosine deaminase acting on RNA (ADAR1), a suppressor of double-stranded RNA-triggered innate immune responses. J. Biol. Chem. *294*, 1710–1720. https://doi.org/10.1074/jbc.TM118.004166.
3. Mannion, N., Arieti, F., Gallo, A., Keegan, L.P., and O'Connell, M.A. (2015). New insights into the biological role of mammalian ADARs; the RNA editing proteins. Biomolecules *5*, 2338–2362. https://doi.org/10.3390/biom5042338.
4. Higuchi, M., Maas, S., Single, F.N., Hartner, J., Rozov, A., Burnashev, N., Feldmeyer, D., Sprengel, R., and Seeburg, P.H. (2000). Point mutation in an AMPA receptor gene rescues lethality in mice deficient in the RNA-editing enzyme ADAR2. Nature *406*, 78–81.
5. Sommer, B., Köhler, M., Sprengel, R., and Seeburg, P.H. (1991). RNA editing in brain controls a determinant of ion flow in glutamate-gated channels. Cell *67*, 11–19.
6. Li, J.B., Levanon, E.Y., Yoon, J.K., Aach, J., Xie, B., Leproust, E., Zhang, K., Gao, Y., and Church, G.M. (2009). Genome-wide identification of human RNA editing sites by parallel DNA capturing and sequencing. Science *324*, 1210–1213. https://doi.org/10.1126/science.1170995.
7. Levanon, E.Y., Hallegger, M., Kinar, Y., Shemesh, R., Djinovic-Carugo, K., Rechavi, G., Jantsch, M.F., and Eisenberg, E. (2005). Evolutionarily conserved human targets of adenosine to inosine RNA editing. Nucleic Acids Res. *33*, 1162–1168. https://doi.org/10.1093/nar/gki239.
8. Nakamura, F., Osborn, T.M., Hartemink, C.A., Hartwig, J.H., and Stossel, T.P. (2007). Structural basis of filamin A functions. J. Cell Biol. *179*, 1011–1025. https://doi.org/10.1083/jcb.200707073.
9. Stulić, M., and Jantsch, M.F. (2013). Spatio-temporal profiling of Filamin A RNA-editing reveals ADAR preferences and high editing levels outside neuronal tissues. RNA Biol. *10*, 1611–1617. https://doi.org/10.4161/rna.26216.
10. Gargiulo, A., Auricchio, R., Barone, M.V., Cotugno, G., Reardon, W., Milla, P.J., Ballabio, A., Ciccodicola, A., and Auricchio, A. (2007). Filamin A is mutated in X-linked chronic idiopathic intestinal pseudo-obstruction with central nervous system involvement. Am. J. Hum. Genet. *80*, 751–758. https://doi.org/10.1086/513321.
11. Hehr, U., Hehr, A., Uyanik, G., Phelan, E., Winkler, J., and Reardon, W. (2006). A filamin A splice mutation resulting in a syndrome of facial dysmorphism, periventricular nodular heterotopia, and severe constipation reminiscent of cerebro-fronto-facial syndrome. J. Med. Genet. *43*, 541–544. https://doi.org/10.1136/jmg.2005.038505.
12. Feng, Y., Chen, M.H., Moskowitz, I.P., Mendonza, A.M., Vidali, L., Nakamura, F., Kwiatkowski, D.J., and Walsh, C.A. (2006). Filamin A (FLNA) is required for cell-cell contact in vascular development and cardiac morphogenesis. Proc. Natl. Acad. Sci. USA *103*, 19836–19841. https://doi.org/10.1073/pnas.0609628104.
13. Zhou, X., Tian, F., Sandzén, J., Cao, R., Flaberg, E., Szekely, L., Cao, Y., Ohlsson, C., Bergo, M.O., Borén, J., and Akyürek, L.M. (2007). Filamin B deficiency in mice results in skeletal malformations and impaired microvascular development. Proc. Natl. Acad. Sci. USA *104*, 3919–3924. https://doi.org/10.1073/pnas.0608360104.
14. Savoy, R.M., and Ghosh, P.M. (2013). The dual role of filamin A in cancer: can't live with (too much of) it, can't live without it. Endocr. Relat. Cancer *20*, R341–R356. https://doi.org/10.1530/ERC-13-0364.
15. Shao, Q.Q., Zhang, T.P., Zhao, W.J., Liu, Z.W., You, L., Zhou, L., Guo, J.C., and Zhao, Y.P. (2016). Filamin A: insights into its exact role in cancers. Pathol. Oncol. Res. *22*, 245–252. https://doi.org/10.1007/s12253-015-9980-1.
16. Bandaru, S., Grönros, J., Redfors, B., Çil, Ç., Pazooki, D., Salimi, R., Larsson, E., Zhou, A.X., Ömerovic, E., and Akyürek, L.M. (2015). Deficiency of filamin A in endothelial cells impairs left ventricular remodelling after myocardial infarction. Cardiovasc. Res. *105*, 151–159. https://doi.org/10.1093/cvr/cvu226.

17. Jain, M., Mann, T.D., Stulić, M., Rao, S.P., Kirsch, A., Pullirsch, D., Strobl, X., Rath, C., Reissig, L., Moreth, K., et al. (2018). RNA editing of Filamin A pre-mRNA regulates vascular contraction and diastolic blood pressure. EMBO J. *37*, e94813. https://doi.org/10.15252/embj.201694813.

18. Jain, M., Weber, A., Maly, K., Manjaly, G., Deek, J., Tsvyetkova, O., Stulić, M., Toca-Herrera, J.L., and Jantsch, M.F. (2022). A-to-I RNA editing of Filamin A regulates cellular adhesion, migration and mechanical properties. FEBS J. *289*, 4580–4601. https://doi.org/10.1111/febs.16391.

19. Lamalice, L., Le Boeuf, F., and Huot, J. (2007). Endothelial cell migration during angiogenesis. Circ. Res. *100*, 782–794. https://doi.org/10.1161/01.RES.0000259593.07661.1e.

20. Wahlberg, E. (2003). Angiogenesis and arteriogenesis in limb ischemia. J. Vasc. Surg. *38*, 198–203. https://doi.org/10.1016/s0741-5214(03)00151-4.

21. Peach, C.J., Mignone, V.W., Arruda, M.A., Alcobia, D.C., Hill, S.J., Kilpatrick, L.E., and Woolard, J. (2018). Molecular pharmacology of VEGF-A isoforms: binding and signalling at VEGFR2. Int. J. Mol. Sci. *19*, E1264. https://doi.org/10.3390/ijms19041264.

22. Le Boeuf, F., Houle, F., Sussman, M., and Huot, J. (2006). Phosphorylation of focal adhesion kinase (FAK) on Ser732 is induced by rho-dependent kinase and is essential for proline-rich tyrosine kinase-2-mediated phosphorylation of FAK on Tyr407 in response to vascular endothelial growth factor. Mol. Biol. Cell *17*, 3508–3520. https://doi.org/10.1091/mbc.e05-12-1158.

23. Savinko, T., Guenther, C., Uotila, L.M., Llort Asens, M., Yao, S., Tojkander, S., and Fagerholm, S.C. (2018). Filamin A is required for optimal T cell integrin-mediated force transmission, flow adhesion, and T cell trafficking. J. Immunol. *200*, 3109–3116. https://doi.org/10.4049/jimmunol.1700913.

24. Akdemir, A.S., Metin Armagan, D., Polat Korkmaz, O., Ozkaya, H.M., Kadioglu, P., Gazioglu, N., Tanriover, N., Dirican, A., and Ozturk, M. (2021). Association between beta arrestin 2 and filamin A gene variations with medical treatment response in acromegaly patients. Minerva Endocrinol. https://doi.org/10.23736/S2724-6507.21.03611-3.

25. Paz-Yaacov, N., Bazak, L., Buchumenski, I., Porath, H.T., Danan-Gotthold, M., Knisbacher, B.A., Eisenberg, E., and Levanon, E.Y. (2015). Elevated RNA editing activity is a major contributor to transcriptomic diversity in tumors. Cell Rep. *13*, 267–276. https://doi.org/10.1016/j.celrep.2015.08.080.

26. Kim, H., Sengupta, A., Glogauer, M., and McCulloch, C.A. (2008). Filamin A regulates cell spreading and survival via beta1 integrins. Exp. Cell Res. *314*, 834–846. https://doi.org/10.1016/j.yexcr.2007.11.022.

27. Czermak, P., Amman, F., Jantsch, M.F., and Cimatti, L. (2018). Organ-wide profiling in mouse reveals high editing levels of Filamin B mRNA in the musculoskeletal system. RNA Biol. *15*, 877–885. https://doi.org/10.1080/15476286.2018.1480252.

28. Licht, K., Kapoor, U., Mayrhofer, E., and Jantsch, M.F. (2016). Adenosine to Inosine editing frequency controlled by splicing efficiency. Nucleic Acids Res. *44*, 6398–6408. https://doi.org/10.1093/nar/gkw325.

29. Hellingman, A.A., Bastiaansen, A.J.N.M., de Vries, M.R., Seghers, L., Lijkwan, M.A., Löwik, C.W., Hamming, J.F., and Quax, P.H.A. (2010). Variations in surgical procedures for hind limb ischaemia mouse models result in differences in collateral formation. Eur. J. Vasc. Endovasc. Surg. *40*, 796–803. https://doi.org/10.1016/j.ejvs.2010.07.009.

30. Aref, Z., de Vries, M.R., and Quax, P.H.A. (2019). Variations in surgical procedures for inducing hind limb ischemia in mice and the impact of these variations on neovascularization assessment. Int. J. Mol. Sci. *20*, E3704. https://doi.org/10.3390/ijms20153704.

31. Overwijk, W.W., and Restifo, N.P. (2001). B16 as a mouse model for human melanoma. Curr. Protoc. Immunol. *Chapter 20*. Unit 20.1. https://doi.org/10.1002/0471142735.im2001s39.

**OMTN, Volume *30***

## Supplemental information

# Filamin A pre-mRNA editing modulates vascularization and tumor growth

**Mamta Jain, Greeshma Manjaly, Kathrin Maly, Margreet R. de Vries, Michael Janisiw, Lisa König, Anne Yaël Nossent, and Michael F. Jantsch**

## Supplemental Information

**Figure S1:**

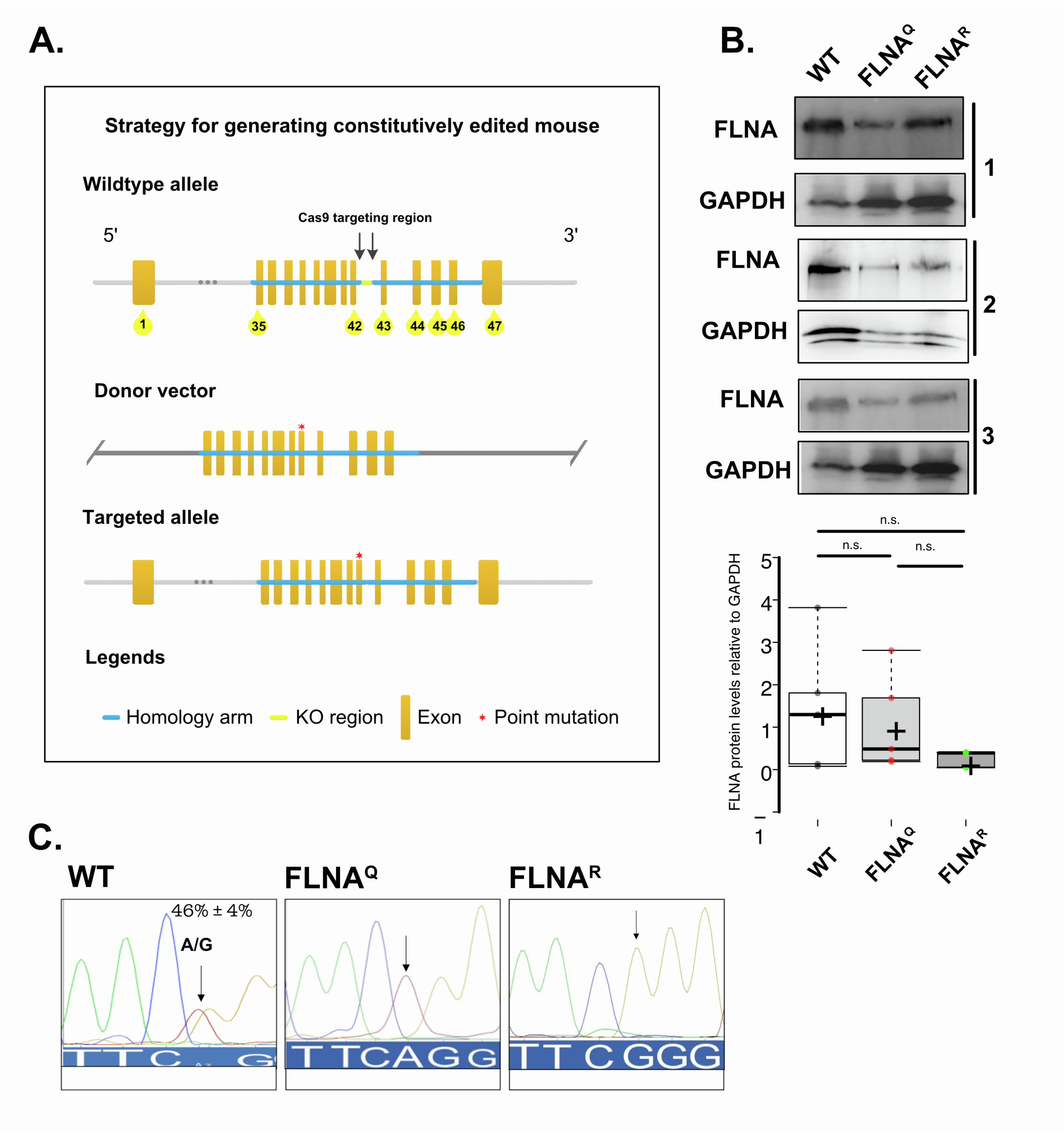


**Figure S1:**

**Generation of transgenic mice expressing constitutively edited FLNA$^R$**

**(A)** Scheme showing the generation of constitutively edited FLNA mouse depicting the wildtype allele, donor DNA and the targeted allele. The diagram also shows the Cas9 targeting region, deleted ECS region and the point mutation to generate a pre-edited version of FLNA. **(B)** Three Western blots showing FLNA expression in wild type (WT), FLNA$^Q$ and FLNA$^R$ lung tissues. GAPDH was used as a loading control. Graph shows the quantification of FLNA expression expressed as fold change difference. Data shown are mean ± SD from five

independent experiments. * P<0.05, ns-non-significant. **(C)** Electropherograms showing FLNA editing from cDNA of lung tissues of WT, $FLNA^Q$ and $FLNA^R$ mice. The site of editing is marked by a black arrow. WT lungs showed an average of 46% FLNA editing.

**Figure S2**

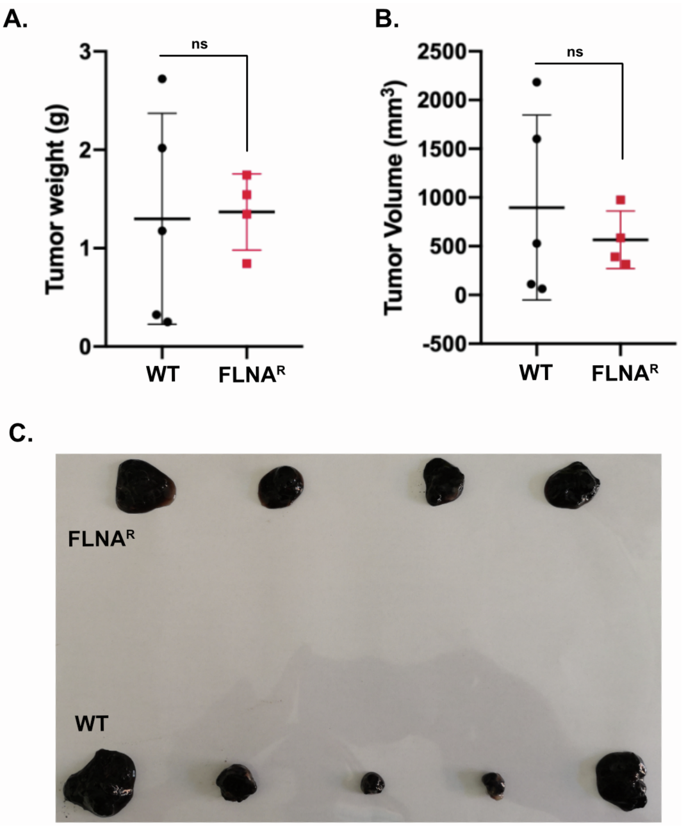


**Figure S2: Comparison of growth of xenografted tumors in wild-type and transgenic mice.** Graphs showing quantification of tumor weight (A) and tumor volume (B) of tumors grown in WT or transgenic mice expressing fully edited $FLNA^R$. The data represents the tumor-weight and volume measurements done on at least 4 mice per genotype. (C) shows raw images of tumors. ns: non-significant.

**Figure S3:**

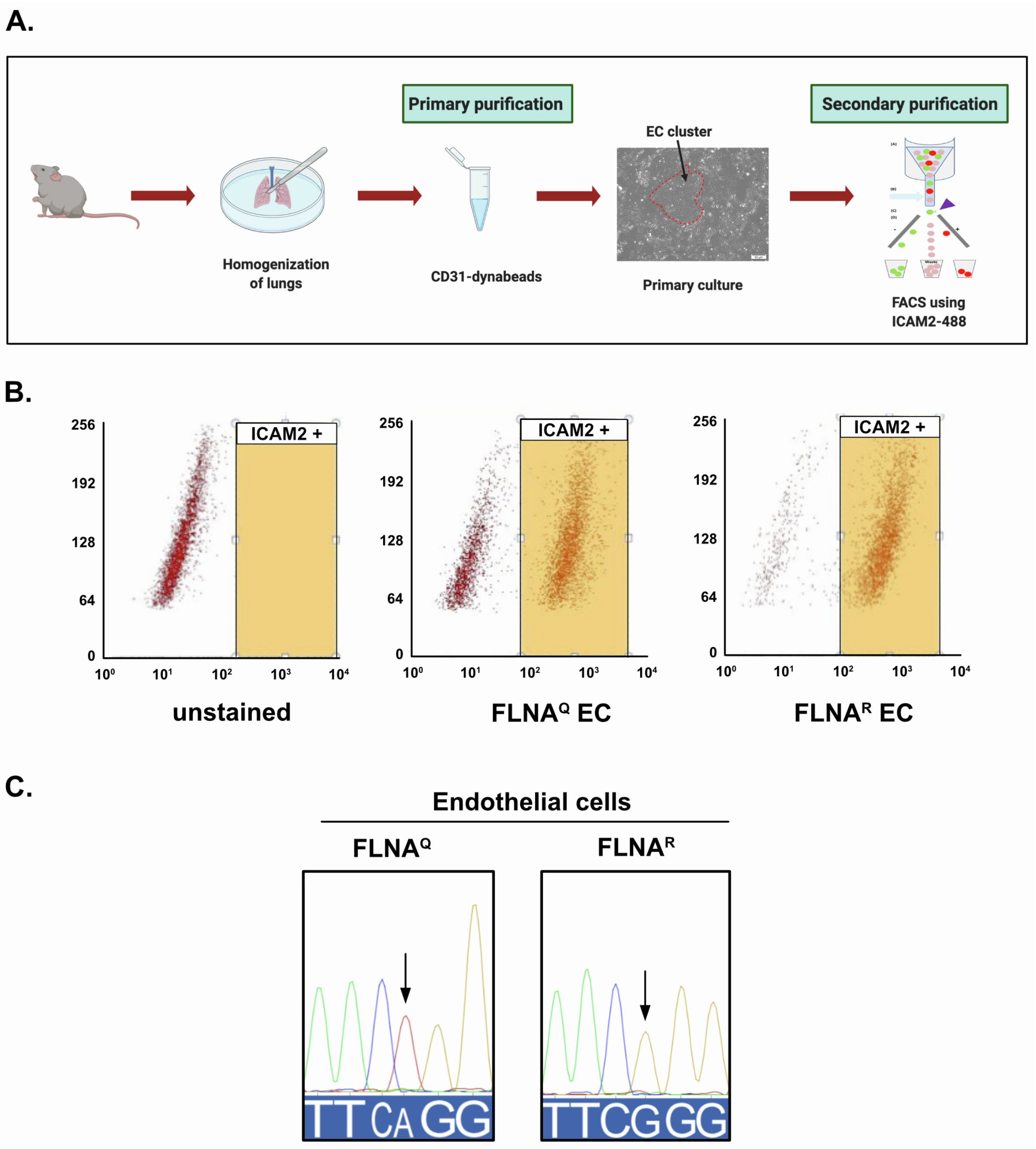


**Figure S3: Purification and characterization of endothelial cells derived from mice expressing unedited FLNA$^{Q}$ and edited FLNA$^{R}$**

**(A)** Scheme depicting the steps involved in primary endothelial cell isolation from lungs of FLNA$^{Q}$ and FLNA$^{R}$ mice. Purification using CD31 dynabeads is followed by a second FACS-sorting step using ICAM2 antibody to obtain a pure endothelial cell population. **(B)** Graphs showing the representative FACS sorting data in unstained, FLNA$^{Q}$ and FLNA$^{R}$ samples. The yellow box represents the sorted endothelial cell population. **(C)** Sanger-sequencing traces showing FLNA editing on cDNA of FLNA$^{Q}$ and FLNA$^{R}$ endothelial cells. The site of editing is marked by a black arrow.

**FigureS4:**

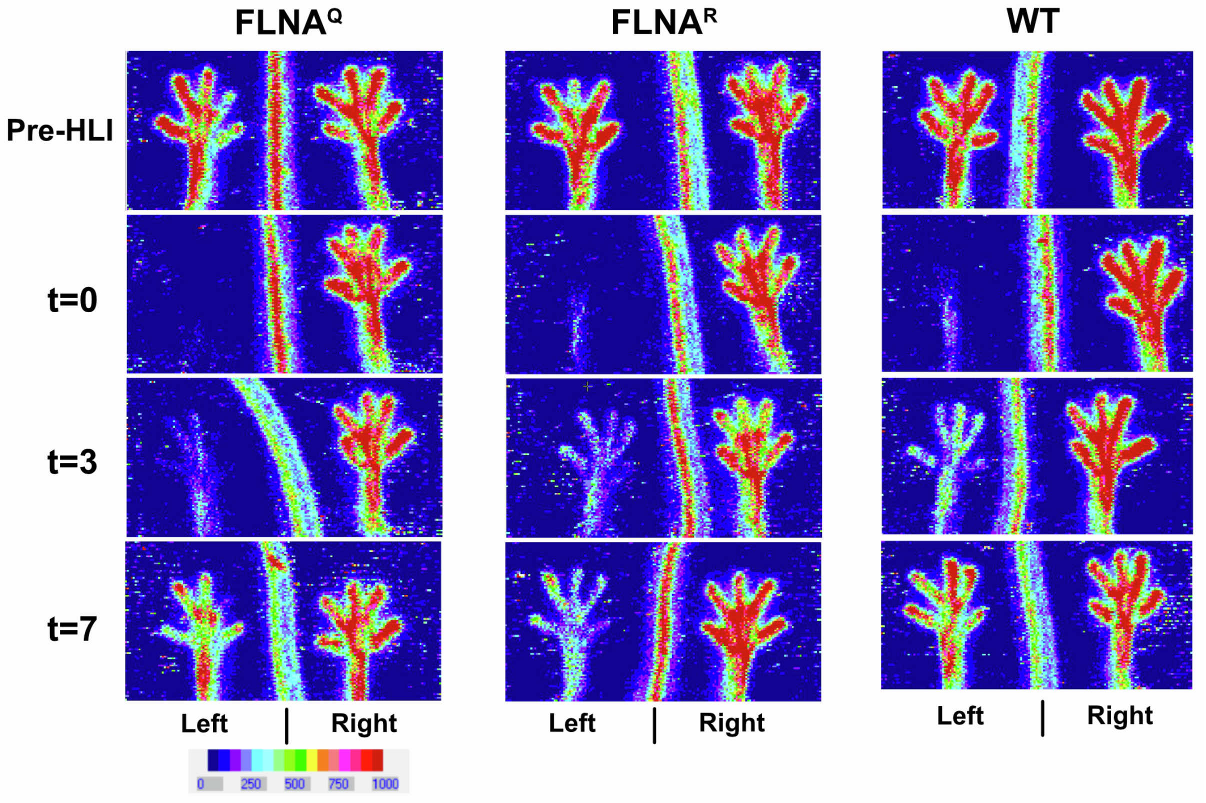


**Figure S4: Reduced blood flow after induced hindlimb ischemia in mice expressing edited FLNA$^R$**

Representative images showing blood flow recovery in the left (post-ischemic) and right control paw in FLNA$^Q$, FLNA$^R$ and WT mice using Laser Doppler perfusion imaging. Paw perfusion was measured in both left (ligated) and right (control) paw pre HLI surgery, immediately after ligation (t=0) and then at 3 days (t=3) and 7 days after surgery (t=7). The shift from blue to red in color indicates an increase in blood flow. Wild-type and transgenic mice expressing unedited FLNA$^Q$ show comparable recovery rates after seven days, while mice expressing edited FLNA$^R$ show much reduced blood flow at 7 days post-surgery. This is an expanded figure of Figure 4 containing images of pre Hind Limb Ischemia (Pre-HLI) and 3 hrs post HLI (t=3). The images of t=0 and t=7 are therefore identical to those of Figure 4.

**Figure S5:**

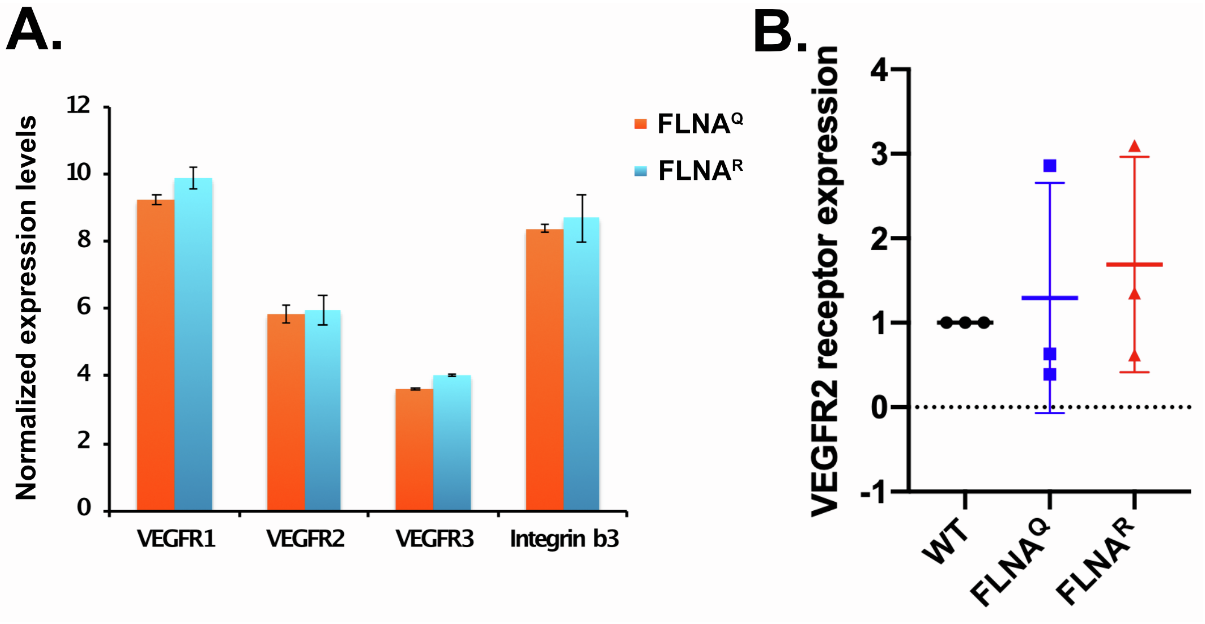


**Figure S5: Expression levels of VEGFR isoforms is unchanged in FLNA^Q and FLNA^R expressing cells and mice.**

(A) Expression levels of VEGFR1, VEGFR2, VEGFR3, and integrin ß1 was compared in endothelial cells expressing unedited FLNA^Q and edited FLNA^R using q-RT-PCR.
(B) Quantification of stainings of xenograft tumors grown in wild-type, FLNAQ and FLNAR expressing mice using an anti-VEGFR2 extracellular domain-specific antibody reveals equal expression of VEGFR2 at steady-state levels.

Figure S6

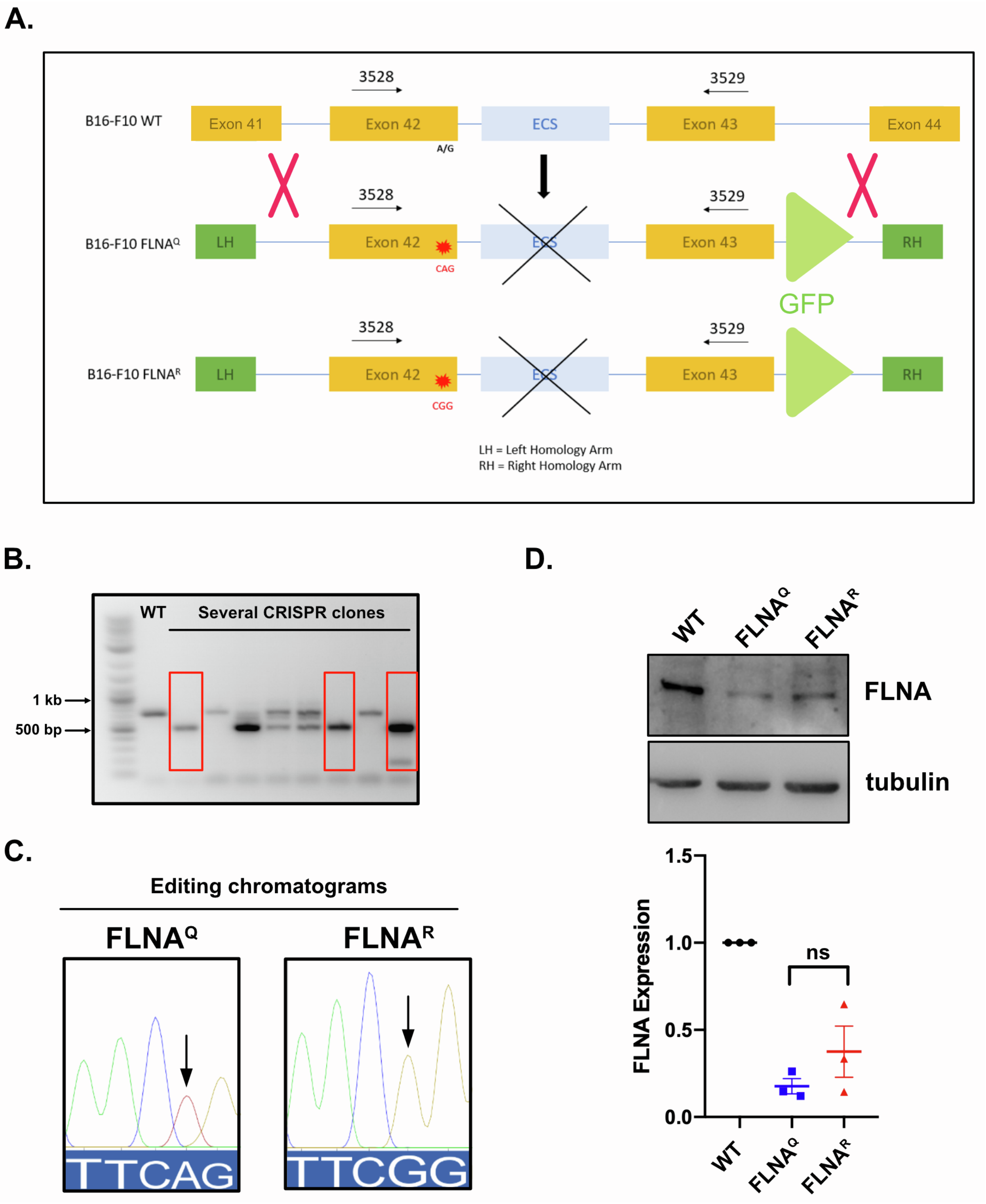


**Figure S6: Generation of B16 cells expressing pre-edited FLNA$^R$ and uneditable FLNA$^Q$**

A) The X-linked *Flna* gene was targeted by CRISPR/Cas9 using two guide-RNAs. As a repair template a region covering parts of the gene was introduced that deleted the editing complementary sequence. In addition, one of the repair templates carried a “pre-edited” CGG codon. B) positive clones were selected by PCR by screening for the deletion of the editing complementary site. C) Expression of unedited FLNA$^Q$ and pre-edited FLNA$^R$ was verified by amplification of cDNA followed by Sanger sequencing of the amplification product. Exclusive expression of unedited FLNA$^Q$ or

edited FLNA$^R$ could be verified. D) Expression of edited and unedited FLNA was tested by western blotting and normalized to tubulin expression. Both modified alleles showed reduced expression when compared to the wild-type alle. However, the difference between edited and unedited FLNA were non-significant (ns).

**Figure S7:**

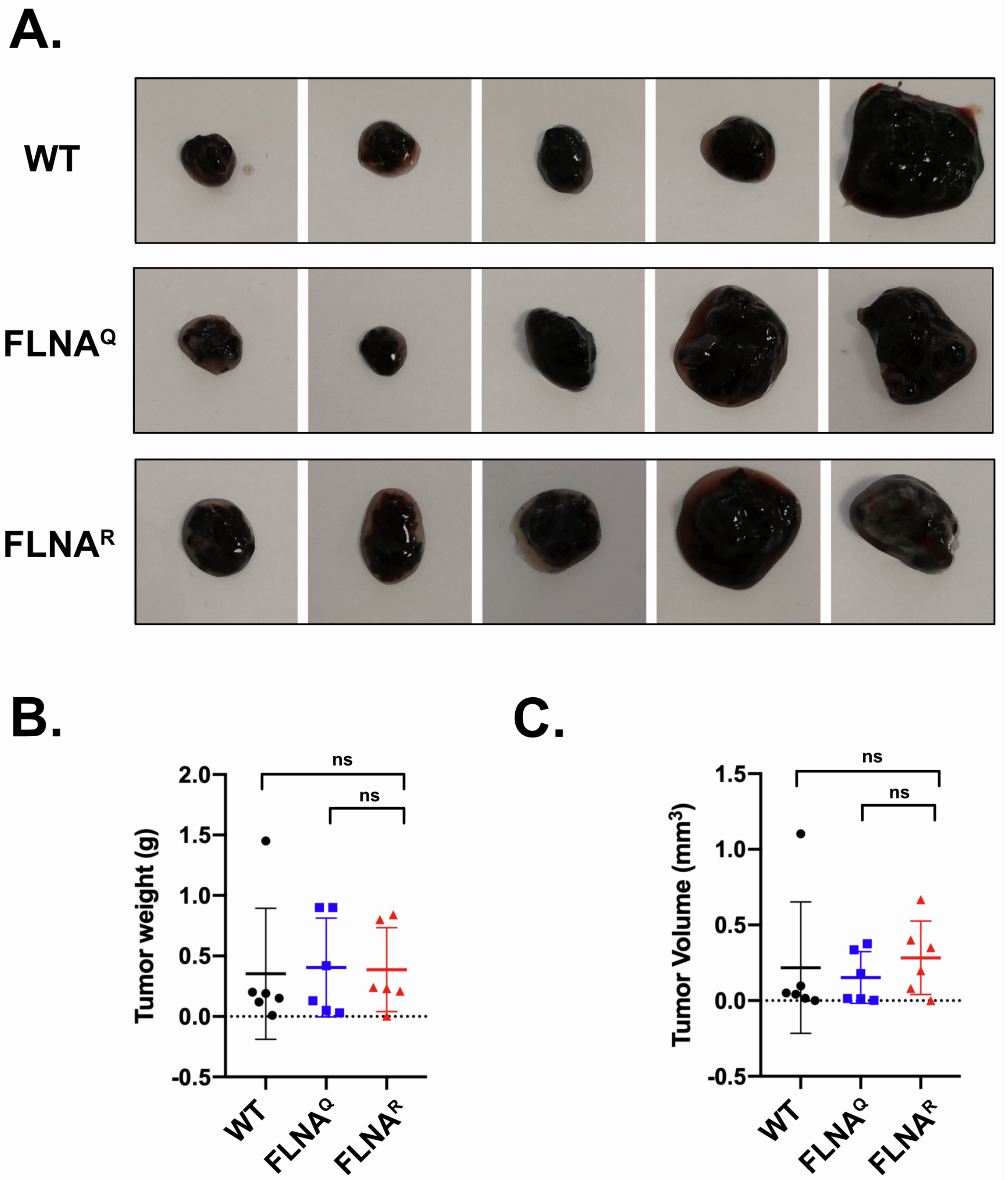


**Figure S7: The editing status of B16 melanoma cells does not affect subcutaneous xenografted tumor growth (A)** Images showing xenograft tumors in WT mice upon subcutaneous injections of WT, FLNA$^{Q}$ and FLNA$^{R}$ B16 CRISPR clones. **(B, C)** Graphs showing the quantification of tumor weight (A) and tumor volume (B) compared amongst WT, FLNA$^{Q}$ and FLNA$^{R}$ B16 CRISPR clones when injected in WT C57Bl6 mice subcutaneously. The data represents the mean ± SD from atleast 12 mice per genotype. ns: non-significant
